# Aspen Plus^®^-Validated CCD–RSM Optimisation of Pressurised Ethanol/Water Extraction for Sustainable Recovery of Antioxidant and Photoprotective Constituents from *Inula salicina* L.

**DOI:** 10.3390/antiox15040466

**Published:** 2026-04-09

**Authors:** Marius Užupis, Michail Syrpas, Andrius Jaskūnas, Petras Rimantas Venskutonis, Vaida Kitrytė-Syrpa

**Affiliations:** 1 Department of Food Science and Technology, Kaunas University of Technology, Radvilėnų Rd. 19, LT-50254 Kaunas, Lithuania; marius.uzupis@ktu.lt (M.U.); michail.syrpas@ktu.lt (M.S.); rimas.venskutonis@ktu.lt (P.R.V.); 2 Department of Physical and Inorganic Chemistry, Kaunas University of Technology, Radvilėnų Rd. 19, LT-50254 Kaunas, Lithuania; andrius.jaskunas@ktu.lt

**Keywords:** *Inula salicina*, pressurised hydroethanol extraction, CCD-RSM optimisation, Aspen Plus^®^ simulation, in vitro antioxidant activity, chlorogenic acid

## Abstract

This study presents an integrated approach for producing antioxidant-rich polar fractions from *Inula salicina* L. via pressurised ethanol/water extraction (PLE-EtOH/H_2_O), optimised by coupling a central composite design and response surface methodology (CCD-RSM) with Aspen Plus^®^ simulation. The effects of PLE temperature, extraction time, and EtOH/H_2_O ratio for yield, total phenolic (TPC) and flavonoid (TFC) content, and Trolox equivalent antioxidant capacity (TEAC) measured in ABTS^•+^-scavenging, cupric ion reducing antioxidant (CUPRAC) and oxygen radical absorbance (ORAC) assays were assessed via a multi-response optimisation approach. Optimal conditions were set at 82 °C, 27 min, and 60% EtOH (*v*/*v*), yielding ~29 g extract per 100 g plant material, characterised by high TPC (227 mg GAE/g), TFC (34 mg QE/g), and TEAC values in the CUPRAC (1473 mg TE/g), ABTS (869 mg TE/g), and ORAC assays (1165 mg TE/g). The TPC and TEAC values of the post-extraction residue were >92% lower than those of unextracted *I. salicina*, confirming efficient recovery of the major portion of antioxidant-active constituents by PLE-EtOH/H_2_O. The high in vitro radical scavenging capacity, reducing power, and photoprotective potential (sun protection factor ~50 at 0.5 mg/mL) of the *I. salicina* extract are consistent with its phenolic-rich composition, with chlorogenic acid (~97 mg/g extract) and its derivatives being the major constituents. The validated Aspen Plus^®^ model closely aligned with the CCD-RSM predictions, supporting process scale-up and energy feasibility and demonstrating an industry-relevant, green-solvent PLE process for producing higher value-added *I. salicina* fractions with potential applications in the food, pharmaceutical, nutraceutical, and cosmetic sectors.

## 1. Introduction

From ancient times to modern-day practices, during which approximately 80% of the global population still relies on traditional, complementary, or alternative medicine, humanity has recognised the therapeutic potential of nature-based treatments and medicinal plants [1]. Nevertheless, even in modern pharmaceutical research, there is a renewed interest in medicinal plants, which have long served as valuable sources of therapeutic compounds and continue to provide a significant reservoir of novel drug candidates. Moreover, nowadays, the role of botanicals has not diminished; on the contrary, it has increased, particularly with the advent of the nutraceutical and functional food era [2]. Among these, plants of the genus *Inula*, part of the Asteraceae family, comprise more than 100 species that are widely distributed globally and are commonly used in traditional medicine in Europe and Asia, with a prominent role in traditional Chinese medicine, demonstrating diverse activities, including antibacterial, cytotoxic, hepatoprotective, anti-inflammatory, and anticancer effects [3,4]. However, only a limited number of these species have been studied in detail, with pharmacological activities experimentally validated and their inclusion in pharmacopoeias documented [4]. Examples include *I. helenium*, traditionally indicated for inflammation-related disorders of the respiratory tract, including cough, bronchial and throat conditions, bronchitis, catarrh, and colds, and is also described as an immunostimulant [5]. *I. britannica* has been used for the management of intestinal disorders and inflammation and has also been described as a carminative, diuretic, laxative, and remedy for hepatitis [6]. *I. viscosa* has been described as a traditional remedy for bronchitis and tuberculosis treatment, as an antiseptic, and expectorant; it is also applied topically to promote wound healing and relieve rheumatic pain [7]. Previous studies of ethnomedicinally important *Inula* species (e.g., *I. helenium*, *I. racemosa*, *I. orientalis*, and *I. britannica*) have identified more than 400 constituents, among which sesquiterpene lactones and a variety of polyphenols, mainly flavonoids (e.g., quercetin, kaempferol, and luteolin) and hydroxycinnamic acid derivatives (e.g., chlorogenic acids), are considered the principal bioactive compounds that contribute to these diverse biological effects [8,9].

Among *Inula* species, *I. salicina*, currently also accepted as *Pentanema salicinum* (L.) [10], and commonly known as Irish fleabane or willow-leaved yellowhead, has been comparatively understudied despite its use in folk medicine for treatment of respiratory infections, skin diseases, catarrh, and seizures. Consequently, its pharmacological properties and biological activities remain insufficiently characterised, and information on its chemical constituents is limited. For example, a recent report on its properties has demonstrated the neuroprotective potential of *I. salicina* extracts, with both aqueous and methanolic extracts exhibiting protective effects against maximal electroshock-induced seizures in mice, and acting as anticonvulsants [11]. In another study, extracts of *I. salicina* grown in Bulgaria were demonstrated to have promising anti-biofilm and quorum-quenching properties [6]. Additionally, its methanolic extract could be utilised in cosmetic formulations due to its antibacterial and sun-protection properties [12]. Phytochemical studies of the chloroform extract of this plant revealed the presence of triterpene alcohols and their 3-O-esters [7], while its methanolic extract was characterised by chlorogenic acid, as well as 3,5, 1,5-, and 4,5-dicaffeoylquinic acids, among 58 identified components [12]. Moreover, one study has investigated the volatile compound composition of *I. salicina* aerial parts, identifying hexahydrofarnesyl acetone as the major essential oil constituent and reporting antibacterial, antinociceptive, and anti-inflammatory activities [13]. Over recent years, substantial effort has been directed toward developing and optimising green, industrially viable extraction processes for recovering high-value phytochemicals with diverse bioactivities from various natural resources for pharmaceutical, nutraceutical, food, cosmetic, and agrochemical applications, while achieving high process efficiency, selectivity, stability of phytochemical composition, and bioactivity. In this context, process-intensified techniques, coupled with environmentally benign solvents, are increasingly implemented to enhance performance and economic feasibility while reducing energy demand and solvent-related hazards. This further supports the transition from conventional solvent-intensive operations to safer, more sustainable downstream processing, aligning with the United Nations Sustainable Development Goals (SDGs) by reducing environmental burdens and improving the overall sustainability of phytochemical production chains [14,15,16]. Pressurised liquid extraction (PLE), also known as accelerated solvent extraction, is listed among these green and sustainable extraction techniques, especially when readily available, non-toxic, and recyclable food-grade solvents such as water and ethanol are used [17,18]. PLE is also increasingly applied in green analytical chemistry, providing higher extraction yields, reduced solvent consumption, lower energy demand, and shorter extraction times, while the optimisation of operating parameters (e.g., temperature, pressure, time, solvent composition, etc.) enables the selective recovery of target constituents from diverse matrices [19,20]. In recent years, numerous studies have demonstrated the suitability of PLE for isolating higher value-added fractions from various plant materials, including medicinal plants. Considering the abundance of bioactive constituents, particularly phenolic compounds, *in I. salicina*, PLE offers several advantages over conventional extraction methods for the sustainable preparation of phenolic-enriched extracts, as elevated temperature and pressure improve solvent penetration into the plant matrix and enhance mass transfer, resulting in more efficient phytochemical recovery, with shorter extraction times and lower solvent use [18].

As medicinal plants are chemically complex matrices, the efficiency and selectivity of PLE can vary substantially with the choice of operating parameters and their experimental ranges. Therefore, for a given plant matrix, the optimisation of extraction conditions by identifying the most influential process variables and their interactions is essential not only to maximise yield but also to minimise the degradation of thermolabile constituents and to obtain fractions with the desired phytochemical profile and functional properties, such as antioxidant capacity, for potential pharmaceutical, cosmetic, or nutraceutical applications. For this purpose, design of experiments (DoEs) approaches, such as central composite design coupled with response surface methodology (CCD–RSM), are frequently applied to model process responses and identify optimal settings [21]. Increasingly, multivariate DoE-based optimisation is favoured over one-factor-at-a-time testing because it enables the efficient determination of optimal operating conditions by simultaneously evaluating the effects and interactions of multiple process variables on multiple response factors with fewer experiments. As plant extracts are inherently complex matrices containing diverse phytochemicals that can respond differently to changes in extraction conditions, this approach is particularly relevant in plant extract manufacturing, as it enables balancing overall process productivity (e.g., yield) with extract quality attributes, such as phytochemical composition and bioactivity indices (e.g., antioxidant activity) [22]. Furthermore, the combined use of CCD-RSM with physical process simulation, e.g., using Aspen Plus^®^, can provide an integrated framework for extraction optimisation that is more informative than either approach alone. For instance, CCD-RSM offers a statistically robust description of how the selected responses vary with operating conditions, identifies significant factor interactions, and enables optimisation within the experimental design space. Aspen Plus^®^ complements this mathematical modelling by translating the experimentally derived relationships into a physicochemical process based on mass-transfer and phase-equilibrium assumptions, while also applying mass and energy balances, thermodynamic models, and unit operation descriptions relevant to process simulation enforcing mass and energy balances, thermodynamic models, and unit operation descriptions suitable for process simulation and scale-up. Therefore, the synergy of these two approaches lies in linking statistical optimisation at laboratory scale with process-level physical modelling, providing a more robust and industrially relevant basis for extraction design and future scale-up [23,24].

Reports describing the use and optimisation of various intensified extraction technologies in *Inula* species are limited and primarily focus on *I. helenium* roots, where ultrasound-assisted extraction, microwave-assisted extraction, and high-pressure homogenization have been applied to isolate phenolic compounds, flavonoids, and sesquiterpene lactones [25,26,27,28,29,30]. In contrast, only a few recent studies have examined other *Inula* species, reporting improved extraction efficiency for optimised ultrasound-assisted extraction of *I. viscosa* [31] and subcritical water extraction of the aerial parts of *I. racemosa* [32] compared with conventional techniques. The scarce reports on *I. salicina* bio-functional characterisation have been conducted solely using time-consuming conventional extraction techniques, often involving hazardous organic solvents, thereby limiting the potential applications of such extracts. To address these gaps and align with industrially relevant process development, this study integrates CCD-RSM optimisation of pressurised ethanol/water extraction (PLE-EtOH/H_2_O) with Aspen Plus^®^ process simulation to approximate larger-scale operations for the recovery of antioxidant-rich fractions from *I. salicina*. A multi-response optimisation was employed to simultaneously evaluate the effects of extraction temperature, time and solvent composition on the extract yield, total phenolic and flavonoid content, and in vitro antioxidant activity in ABTS^•+^-scavenging, CUPRAC, and ORAC assays, further complemented by in vitro photoprotective properties by phytochemical characterisation using ultraperformance liquid chromatography–electrospray tandem mass spectrometry (UPLC-ESI/MS^2^) analysis of the resulting extract under optimal PLE-EtOH/H_2_O conditions. To the best of our knowledge, this study provides one of the first integrated experimental and process-modelling approaches, demonstrating the potential of an optimised and scalable PLE process to obtain higher value-added *I. salicina* fractions suitable for pharmaceutical, nutraceutical, and cosmetic applications. The proposed extraction process aligns with several SDGs by advancing scalable, resource-efficient recovery of health-oriented bioactives (SDG 3) through green technologies (SDG 9), thereby strengthening sustainable agri-food value chains and related economic opportunities (SDG 8).

## 2. Materials and Methods

### 2.1. Plant Material and Reagents

Dried aerial parts of *I. salicina* (consisting predominantly of leaves with minor amounts of stems and inflorescences), further abbreviated as plant material (PM), were acquired from UAB “Jadvygos žolės” (collected in 2023). Before extraction, the PM was ground in a ZM 200 laboratory cyclone mill (Retsch, Haan, Germany) to a particle size of 0.5 mm and stored at ambient temperature in a glass container in a dark, well-ventilated place. Analytical and HPLC-grade solvents, reagents, and standards were used for the experiments (Supplementary Material, S1).

### 2.2. Preparation of I. salicina Extracts by Soxhlet and PLE Extraction

Soxhlet extraction with ethanol (further abbreviated as SE-EtOH) was carried out using 3.000 ± 0.001 g of PM in an automated Soxhlet extractor (EZ100H, Behr Labor-Technik, Düsseldorf, Germany) with 100 mL of ethanol, under reflux at 78 °C for 6 h.

PLE was performed with ethanol (further abbreviated as PLE-EtOH), water (further abbreviated as PLE-H_2_O), or hydroethanolic mixtures (further abbreviated as PLE-EtOH/H_2_O) using an accelerated solvent extractor ASE 350 (Dionex, Sunnyvale, CA, USA). For each extraction, 3.000 ± 0.001 g of PM was mixed with diatomaceous earth (1:1, *w*/*w*) and placed in a 34 mL stainless steel extraction cell. Diatomaceous earth was used as an inert dispersing agent to minimise particle aggregation, increase the sample’s accessible surface area to the solvent, and fill void spaces in the extraction cell, thereby reducing solvent consumption during extraction. The extraction conditions were: 70 °C for 45 min (3 × 15 min cycles) for PLE-EtOH, 110 °C for 45 min (3 × 15 min cycles) for PLE-H_2_O, and variable conditions for PLE-EtOH/H_2_O, including temperatures of 40–100 °C, extraction time of 15–45 min (3 cycles × 5–15 min), and an EtOH/H_2_O ratio of 20–80% (*v*/*v*). A solid-to-liquid ratio of 1:10 (*w*/*v*), a pressure (P) of 10.3 MPa, a preheating time of 5 min, a 100% cell flush volume, and a 120 s nitrogen purge were maintained constant for all extractions.

After extraction, EtOH was removed using a Rotavapor (V-850 R-210, Büchi, Flawil, Switzerland) and H_2_O by freeze-drying (−50 °C, 0.5 mbar). Before storage, the resulting extracts (further abbreviated as E) from SE-EtOH, PLE-EtOH, PLE-H_2_O, and PLE-EtOH/H_2_O were exposed to a nitrogen flow for 5 min and further stored at −18 °C until analysis. Extraction yields were determined gravimetrically (±0.001 g) and expressed as g/100 g PM, based on triplicate experiments.

### 2.3. Phytochemical Composition Analysis by UPLC-ESI/MS^2^

The ultraperformance liquid chromatography–electrospray tandem mass spectrometry (UPLC-ESI/MS^2^) analysis was performed on a Shimadzu i-series LC-2060 (Shimadzu Corporation, Kyoto, Japan) coupled to an LC-MS 8045 (Shimadzu Corporation, Kyoto, Japan) mass spectrometer. For UPLC-ESI/MS^2^ analysis, the extract obtained under optimised PLE-EtOH/H_2_O conditions was dissolved in ultrapure H_2_O to 10 mg/mL, diluted tenfold (1 mg/mL) and filtered through a 0.45 µm hydrophilic PTFE membrane filter. The UPLC column was a Discovery HS F5-3 (2.1 mm I.D. × 150 mm L., 3 μm) (Merck Group, Darmstadt, Germany). The mobile phases were solvent A: ultrapure H_2_O containing 0.1% (*v*/*v*) formic acid; and solvent B: methanol containing 0.1% (*v*/*v*) formic acid. The gradient profile was regulated as follows: 20% B at 0–1 min, 50% B at 1–18 min, 80% B at 18–22 min, 95% B at 22–23 min, 95% B at 23–25 min, 20% B at 25–26 min, and 20% B at 26–30 min. The following settings were used: injection volume (5 μL), mobile phase flow rate (0.6 mL/min), column temperature (40 °C), desolvation temperature (526 °C), desolvation line temperature (250 °C), drying gas flow (10 L/min), heat block temperature (400 °C), heating gas flow (10 L/min), interface temperature (300 °C), and nebulising gas flow (2 L/min).

For the MS analysis, electrospray ionisation (ESI) was employed in negative or positive mode. In a set of preliminary experiments, precursor ions of compounds present in PLE-EtOH/H_2_O extract were identified; in the next step, a product ion scan was performed with the triple quadrupole mass spectrometer LC-MS 8045 to analyse the fragment ions from the selected precursor ions. Precursor–product ion pairs, which agreed with the previously published data, were selected for further analysis in the multiple reaction monitoring (MRM) mode. Compounds were identified according to the Metabolomics Standards Initiative (MSI) guidelines [33] for qualitative annotation. The compounds matched to authentic reference standards by both retention time and MRM transitions were classified as Level 1 (identified compounds). Compounds annotated without reference standards, based on accurate mass, MS/MS fragmentation patterns, and comparison with published literature data, were classified as Level 2 (putatively annotated compounds; tentative identification). The compounds without sufficient evidence for exact structural annotation were assigned to a broader chemical class and classified as Level 3 (putatively characterised compound classes; tentative identification). Collision energies of the tentatively identified compounds were selected based on the available previously reported values for these substances. For quantification purposes, an external calibration curve of the chlorogenic acid standard was constituted by plotting the quantifier ion peak areas versus the nominal concentrations. The concentrations that provided signal-to-noise ratios of ≥3 and ≥10 established the limit of detection (LOD) and quantification (LOQ) values. The quantities were reported in mg/g extract (further abbreviated as E) and PM based on triplicate experiments.

### 2.4. In Vitro Antioxidant and Photoprotective Properties of I. salicina Extracts and Plant Material

Following the modified protocols of Singleton et al. [34], Vongsak et al. [35], Apak et al. [36], Re et al. [37] and Prior et al. [38], in vitro antioxidant capacity of *I. salicina* SE-EtOH, PLE-EtOH, PLE-H_2_O, and PLE-EtOH/H_2_O extracts were assessed as total phenolic content (TPC, expressed as gallic acid equivalents, mg GAE/g E and PM), flavonoid content (TFC, expressed as guercetin equivalents, mg QE/g E and PM), and Trolox equivalent antioxidant capacity (TEAC, mg TE/g E and PM) in cupric ion reducing antioxidant (TEAC_CUPRAC_), ABTS^•+^-scavenging (TEAC_ABTS_) and oxygen radical absorbance (TEAC_ORAC_) assays (Supplementary Material, S2). TPC, TEAC_ABTS_ and TEAC_ORAC_ values of *I. salicina* before and after extraction were determined following the QUENCHER approach [39] and using 10 mg of sample (solid dilutions in microcrystalline cellulose) or cellulose (blank), as described elsewhere by our research group [40,41].

For the photoprotective properties determination, the absorbance of *I. salicina* extracts (5–1000 µg/mL) and chlorogenic acid (0.5–97 µg/mL) was measured from 200 to 800 nm at 1 nm intervals, covering the UV-A (315–400 nm) and UV-B (280–315 nm) ranges. The sun protection factor (SPF) and UV-B absorption (%) were calculated as previously described elsewhere [42,43].

All spectrophotometric measurements were performed in quadruplicate using a GENESYS 150 UV–vis spectrophotometer (Thermo Fisher Scientific, Waltham, MA, USA) and a FLUOstar Omega reader (BMG Labtech, Offenburg, Germany).

### 2.5. Modelling of PLE with CCD-RSM and Aspen Plus^®^

#### 2.5.1. Experimental Design

CCD-RSM was used to determine the effect of PLE-EtOH/H_2_O temperature (T, 40–100 °C), extraction time (τ, 15–45 min), and EtOH/H_2_O ratio (20–80%, *v*/*v*) on several response factors (RFs): extraction yield (g/100 g PM), TPC (mg GAE/g E and PM), TFC (mg QE/g E and PM), TEAC_CUPRAC_, TEAC_ABTS_, and TEAC_ORAC_ (mg TE/g E and PM). Design-Expert 13.0.5.0 software (Stat-Ease Inc., Minneapolis, MN, USA) was used to generate a face-centred CCD with 20 experimental runs and to perform the subsequent RSM analysis for each RF. The statistical significance and suitability of the CCD models built for each RF were assessed using the Student’s test (*p*-value) at the 5% probability level (*p* < 0.05), along with the ‘lack of fit’ coefficient and the Fisher test value (F-value), as previously described elsewhere by our research group [40,41,42,44].

#### 2.5.2. Modelling with Aspen Plus^®^

As previously described by Nagybákay et al., with some modifications [44], Aspen Plus^®^ V12 (Aspen Technology, Bedford, MA, USA) was used to develop a physical model for the PLE-EtOH/H_2_O process of *I. salicina*, designed according to the flow streams and equipment models presented in Table S1 (Supplementary Material). PM was modelled as a mixture of polar constituents, primarily flavonoids (represented by quercetin in the model) and phenolic acids (represented by gallic acid), which were considered as part of a broader group of EtOH/H_2_O-soluble antioxidants (represented by Trolox), present within the insoluble lignocellulosic matrix that contributes little to extraction and is therefore treated in Aspen Plus as a nonconventional solid. The PM stream was modelled as a mixture of four components, with initial amounts calculated from the maximum CCR-RSM experimental values, assuming that approximately 10% of each component remains unextracted in the insoluble matter. As indicated in Table S1 (Supplementary Material), the EtOH/H_2_O-soluble fraction was set at 322 mg/g PM (0.322 kg/h), comprising 12 mg/g PM (0.012 kg/h) quercetin, 80 mg/g PM (0.08 kg/h) gallic acid, and 230 mg/g PM (0.23 kg/h) Trolox; the remaining 678 mg/g PM (0.678 kg/h) was considered insoluble matter. The extraction model also incorporated particle size distribution (PSD), with particles ranging from 0.1 to 1 mm, divided into five fractions using a logarithmic method and populated according to a normal distribution (D50 of 0.5 mm, standard deviation of 0.2 mm). The thermodynamic properties and parameters of the selected compounds (quercetin, gallic acid, Trolox) were evaluated using the NIST ThermoData Engine. The non-random two-liquid (NRTL) activity coefficient model was used to calculate the thermodynamic activities of these compounds, to estimate their phase equilibria and binary interactions in the non-ideal liquid phase. The extraction proceeds isothermally and is governed by rate-controlled reactions, described as follows:

(1) X(solid phase) → X (liquid phase)

where X: quercetin, gallic acid, and Trolox.

To evaluate process upscalability, 1 kg/h of PM at 20 °C and 1 atm was supplied to the extractor along with 21.04 kg/h of EtOH/H_2_O at the optimal ratio (60/40% *v*/*v*), with the solvent first pressurised to 10.3 MPa by the pump and then preheated in the heater to target temperatures between 40 and 100 °C. The target components are extracted using an EtOH/H_2_O mixture, and the resulting extract-containing hydroethanolic solution is supplied to the evaporator, where both solvents are vaporised, and the extract is collected at the bottom of the evaporator after separation (Supplementary Material, Figure S1).

### 2.6. Statistical Analysis

Microsoft Excel 2019 and GraphPad Prism 10.4.0 software (GraphPad Software, Boston, MA, USA) were used to calculate mean values and standard deviations, to evaluate differences between means with significant differences (*p* < 0.05 in a one-way ANOVA or an unpaired *t*-test) and to calculate Pearson’s correlation coefficients between the PLE-EtOH/H_2_O extract yield, TPC, TFC, and TEAC values.

## 3. Results and Discussion

### 3.1. Extraction Efficiency of Intensified Versus Conventional Extraction Techniques for Polar Constituent Isolation from I. salicina

In the first part of this study, the effectiveness of PLE for isolating polar antioxidant constituents from *I. salicina* was evaluated in comparison with conventional Soxhlet extraction, both widely used as intensified and conventional solid–liquid extraction methods for laboratory and industrial-scale applications, respectively [14,19,24]. PLE was conducted at 70–100 °C for 45 min using EtOH, H_2_O, and mixtures thereof (50:50 to 70:30, *v*/*v*; Table 1), selected as green, bio-based, environmentally friendly, and food-, cosmetic-, and pharmaceutical-grade solvents [18,20]. According to the European Medicines Agency guideline on residual solvents (EMA/CHMP/ICH/82260/2006) and EU Directive 2009/32/EC, EtOH is an acceptable extraction solvent for producing components and ingredients from a wide range of raw materials for the food and pharmaceutical sectors under good manufacturing practice; moreover, H_2_O is inherently compliant for such applications and, when combined with EtOH, enables a safe, industry-compatible solvent system for plant extract production. The selected PLE temperature and extraction time were chosen to remain within the upper limits typically recommended for recovering polar antioxidant compounds from botanical matrices, as temperatures above 100 °C, particularly when combined with prolonged extraction times, are reported to adversely affect the yield and bioactivity of thermolabile constituents [19,45]. To assess the influence of the extraction method on in vitro antioxidant capacity, TPC and TEAC values were determined in the ABTS^•+^-scavenging and CUPRAC assays (Table 1), which are widely used to evaluate the antioxidant potential of plant materials. TPC provides a spectrophotometric estimate of total phenolic content, which serves as an indirect indicator of antioxidant potential, since these phytochemicals are primary contributors to radical-scavenging and reducing activities in plant materials. The ABTS^•+^-scavenging assay quantifies (relative to the standard antioxidant Trolox) the ability of hydrophilic and moderately lipophilic antioxidants in extracts to neutralise the ABTS^•+^, reflecting the electron- or hydrogen-donating capacity to stabilise reactive species. The CUPRAC assay measures the ability of extract constituents to reduce copper(II) to copper(I) ions, indicating their electron-donating and metal-reducing properties and providing a complementary assessment of overall antioxidant potential [46,47].

The results in Table 1 show that PLE with neat EtOH at 70 °C for 45 min yielded 11.6 g/100 g PM of polar fraction, slightly exceeding the amount obtained after 6 h of conventional Soxhlet extraction. This substantial reduction in extraction time was also accompanied by up to a 2-fold increase in the TPC and TEAC values, expressed both per gram of E and PM. Overall, information on both conventional and intensified extraction methods for *I. salicina* is scarce, with only one study reporting methanolic maceration of the aerial parts at room temperature for 24 h that yielded ~8 g polar extract per 100 g PM [12] and only a few reports focusing on isolating non-polar constituents [13,48]. Previously, Ceylan et al. reported 5–14% yields for methanolic extracts obtained by conventional overnight maceration at room temperature from the aerial parts of eleven Turkish *Inula* species; however, *I. salicina* was not included in their study [49]. Soxhlet extraction using methanol and ethanol has been described for *I. viscosa* leaves [50], a mixture of the aerial parts of *I. germanica* [51], and various anatomical parts (roots, stems, leaves, and inflorescences) of *I. helenium* [52]; however, the corresponding extraction yields were not reported.

Although Soxhlet extraction with solvents of varying polarities is an established technique at both laboratory and industrial scales, it requires prolonged extraction (typically 6–24 h) at the boiling point of the solvent, increasing the risk of thermolabile compound degradation. Additionally, it raises health, environmental, and economic concerns due to substantial solvent consumption, which could be mitigated by applying intensified extraction technologies, such as PLE [53]. Recently, PLE was reported to have a lower environmental footprint than conventional Soxhlet extraction for the recovery of antioxidant-rich fractions from rosemary leaves [54] and beet wastes [55]. Similarly, Porto et al. demonstrated that PLE outperforms classical maceration both environmentally and economically for producing antioxidant and neuroprotective extracts from orange by-products, with markedly lower global warming potential (3.42 versus 18.8 kg CO_2_ eq), higher greenness score (0.64 vs. 0.53), and better economic performance due to lower costs, higher returns, and a shorter payback time [56].

As outlined in Table 1, incorporating from 30 to 50% (*v*/*v*) of H_2_O into EtOH during PLE further augmented extraction yields by approximately 2.5-fold to an average of 28.5 g/100 g PM, also significantly improved TPC recovery (averaging 73 mg GAE/g PM) and TEAC values (averaging 391 and 254 mg TE/g PM in the CUPRAC and ABTS assays, respectively). Pressurised extraction with the most polar solvent tested (H_2_O), applied at 100 °C to maintain subcritical conditions, resulted in the highest overall yield (additional ~24% increase as compared to the hydroethanolic extracts) and increased TEAC and TPC values per g of PM, although it did not lead to additional improvements in ABTS^•+^-scavenging activity. Despite the discussed differences in antioxidant activity when expressed per PM, the activities were similar when standardised per gram of E, with TPC values ranging from 229 to 263 mg GAE/g E, TEAC_CUPRAC_ from 1332 to 1555 mg TE/g E, and TEAC_ABTS_ from 823 to 952 mg TE/g E. Taken together, these results indicate that yields, TPC and TEAC values of PLE-EtOH/H_2_O were comparable to, or only slightly lower than those obtained with PLE-H_2_O. Moreover, hydroethanolic extracts showed stronger UV absorbance across the UV-C (200–280 nm), UV-B (280–315 nm), and UV-A (315–400 nm) regions than neat EtOH or H_2_O-derived extracts at the same concentration (50 µg/mL) (Supplementary Material, Figure S2), indicating greater photoprotective potential thereof. In addition, extraction with hydroethanolic mixtures provides tunable solvent polarity, facilitating the solubilisation of a broader range of moderately polar and highly polar antioxidants at substantially lower temperatures (70 versus 100 °C), thereby reducing the risk of thermolabile compound degradation, lowering energy demand, and improving overall process sustainability. Accordingly, the next stage of this study was focused on optimising PLE with EtOH/H_2_O mixtures to obtain hydrophilic antioxidant-rich extracts with enhanced photoprotective potential from *I. salicina*, while maximising bioactive constituent recovery and overall process efficiency.

### 3.2. Modelling and Multi-Response Optimisation of PLE-EtOH/H_2_O Parameters by CCD-RSM

CCD-RSM was selected to assess the impact of PLE temperature (T, 40–100 °C), time (τ, 15–45 min), and EtOH/H_2_O ratio (20–80%, *v*/*v*) on several response factors (RF), namely PLE extract yield, TPC, TFC, and TEAC values in CUPRAC, ABTS, and ORAC assays (Table 2), enabling the simultaneous assessment of multiple factors and their interactions with fewer experiments than a classical one-factor-at-a-time optimisation approach [22]. In addition, multi-response optimisation that combines yield with compositional and bioactivity-related RFs (e.g., TPC, TFC, and TEAC values from various antioxidant assays), considering both PM and E-based responses, provides better insight by capturing both extraction efficiency and the functional properties of the extract. Other factors that could also influence PLE performance were kept constant throughout this study. Namely, pressure was maintained at 10.3 MPa in all experiments, which falls within the range commonly reported for PLE (10–15 MPa). At this pressure, the main function is to keep the solvent in the liquid state at elevated temperatures, while temperature, solvent composition, and extraction time generally exert a stronger effect on extraction efficiency [19]. The sample particle size (0.5 mm) and the solid-to-liquid ratio (1:10, *w*/*v*) were set based on the loading capacity of the extraction cell, previous findings from our research group [42,44], and the consideration that smaller particles (e.g., 0.2 mm) may promote bed compaction and clogging of the extraction system, thereby negatively affecting extraction performance and reproducibility.

The RF values on a PM basis (Table 2) show that the extraction yield (RF_1_, 15.9–30.8 g/100 g PM), TPC (RF_3_, 38.2–72.6 mg GAE/g PM), TEAC_CUPRAC_ (RF_5_, 211.1–420.5 mg TE/g PM), TEAC_ABTS_ (RF_7_, 127.7–265.8 mg TE/g PM), TEAC_ORAC_ (RF9, 181.8–338.8 mg TE/g PM), and TFC (RF_11_, 5.0–10.6 mg QE/g PM) significantly increased by up to two-fold across the defined PLE operating region. The variation from minimum to maximum values per E-basis was lower, ranging from 23% for TEAC_ORAC_ to 43% for TEAC_ABTS_, and 33–37% for other RFs, with TPC (RF_2_) at 203.3–269.4 mg GAE/g E, TEAC_CUPRAC_ (RF_4_) at 1124.0–1541.8 mg TE/g E, TEAC_ABTS_ (RF_6_) at 647.9–927.1 mg TE/g E, TEAC_ORAC_ (RF_8_) at 982.9–1206.0 mg TE/g E, and TFC (RF_10_) at 27.6–37.2 mg QE/g E (Supplementary Materials, Table S2). For most models, particularly those describing PM-based responses, the predicted values were in close agreement with experimentally obtained ones (Supplementary Materials, Figure S3). On a plant-material basis, all RFs were strongly and positively correlated, with the extraction yield (RF_1_) showing high correlations (>0.73) with TPC (RF_3_), TFC (RF_11_), and in vitro antioxidant capacity indices (RF_5_, RF_7_, RF_9_), most of which were significant at *p* < 0.0001 (Supplementary Materials, Table S3). On an E-basis, RFs also showed positive and mostly significant correlations with TPC (RF_2_), correlating strongly with TEAC_CUPRAC_ (RF_4_) and TFC (RF_10_) and moderately with TEAC values in the ABTS and ORAC assays (RF_6_ and RF_8_, respectively) (Supplementary Materials, Table S3), indicating that phenolic and flavonoid constituents are major contributors to the measured antioxidant activity of *I. salicina* PLE-EtOH/H_2_O extracts.

As indicated by the fit statistics and the ANOVA results (Supplementary Materials, Tables S4 and S5), initially, all RFs of *I. salicina* PLE-EtOH/H_2_O were fitted using a quadratic model, with the Fisher (F) values ranging from 4.32 to 73.39 and generally meeting the commonly accepted adequate precision threshold (>4), confirming an adequate signal-to-noise ratio. To improve predictive performance, all models were refitted by eliminating non-significant linear and quadratic interaction terms, resulting in most RFs being best described by a modified quadratic model (RF_1_, RF_3_, RF_4_–RF_7_, RF_9_, RF_11_), RF_8_ and RF_10_ by using a two-factor interaction (2FI) model, and RF_2_ being most accurately represented by a linear model. The modifications consistently increased the F-values (6.80–114.23) and adequate precision and also improved the agreement between the adjusted and predicted R^2^ values (ΔR^2^ < 0.05 for most RFs). For RF_2_, RF_10_, and RF_11_, ΔR^2^ was also substantially reduced to values below 0.15, whereas before modification it ranged from 0.26 to 0.37, exceeding the commonly accepted threshold of 0.2. However, the statistical strength and predictive capability varied markedly among RFs, with the models expressed on the PM basis (RF_1_, RF_3_, RF_5_, RF_7_, RF_9_, and RF_11_) generally showing higher precision than those expressed on the E-basis (RF_2_, RF_4_, RF_6_, RF_8_ and RF_10_). For example, the reduced quadratic model of RF_1_ (extraction yield) was highly robust, exhibiting the highest F value, adequate precision (~36), and R^2^ values > 0.94, whereas the model of RF_8_ (TEAC_ORAC_, mg TE/g E) was the weakest, showing the lowest R^2^ and adequate precision values and the largest discrepancy between adjusted and predicted R^2^ (ΔR^2^~0.4) (Supplementary Materials, Table S4). Differences in model precision and predictive capacity among various RFs (e.g., yield, antioxidant capacity indices, and phytochemical composition) have also been reported in PLE optimisation studies for other plant materials, e.g., spent hops [44] and birch leaves [42]. In this context, the most robust models are commonly obtained for extraction yield, as it is mainly governed by bulk mass transfer and solubility phenomena that are often strongly and positively influenced by PLE temperature and time. In contrast, TPC, TFC, and TEAC values depend not only on extraction efficiency but also on the activity and stability of specific phytochemicals, introducing additional variability in the models.

**Figure 1:**
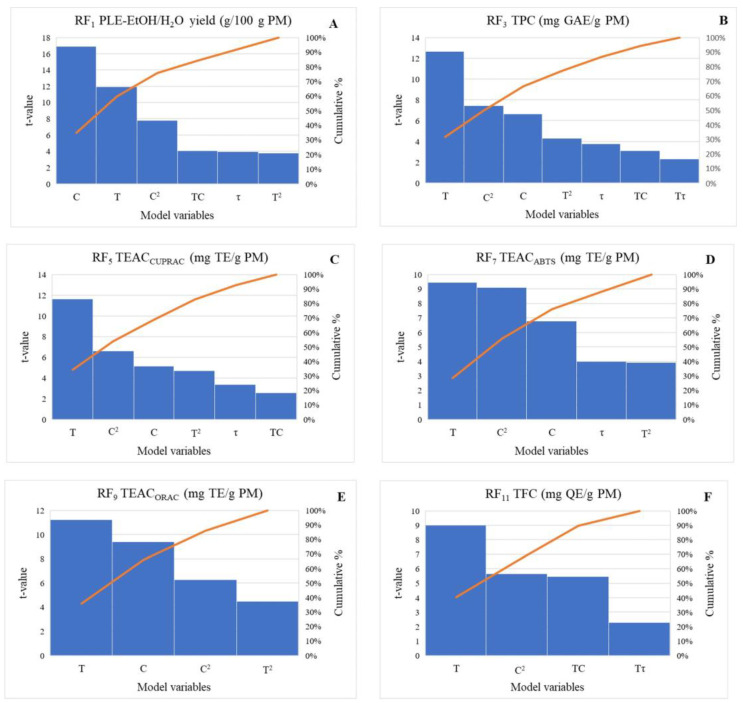
Pareto charts for the main effects of PLE-EtOH/H_2_O temperature (T, °C), time (*τ,* min), and the EtOH/H_2_O ratio (C, % *v*/*v*) and their interactions in *I. salicina*: (**A**) PLE-EtOH/H_2_O extract yield (g/100 g PM); (**B**) total phenolic content (TPC, mg GAE/g PM); (**C**) cupric ion reducing antioxidant (TEAC_CUPRAC_, mg TE/g PM); (**D**) ABTS^•+^-scavenging capacity (TEAC_ABTS_, mg TE/g PM); (**E**) oxygen radical absorbance capacity (TEAC_ORAC_, mg TE/ g PM); (**F**) total flavonoid content (TFC, mg QE/g E).

The first- and second-order polynomial regression equations in actual and coded factors for each RF, demonstrating the relations between the dependent and independent variables (T, τ and C) in the modified models, are provided in Table S6 (Supplementary Materials). Equations in coded factors use standardised variables (scaled to −1, 0, +1) and enable direct comparison of the direction and magnitude of factor effects within the selected PLE operability region, whereas equations in actual factors are expressed in real units of T, τ, and the EtOH/H_2_O ratio and can be used for the development of physical models for PLE extraction, e.g., using Aspen Plus^®^. The ANOVA analysis of the modified models highlights the multifactorial control of the RFs by T, τ, the EtOH/H_2_O ratio, as well as their linear interaction and quadratic terms, with F-values extending from a minimum of 0.2041 for the influence of τ on RF_11_ to a maximum of 285.62 for the main effect of the EtOH/H_2_O ratio on RF_1_ (Supplementary Materials, Table S5). The obtained data indicate that PM-based responses (RF_3_, RF_5_, RF_7_, RF_9_, RF_11_) are mainly governed by extraction T, whereas the RFs expressed per gram of E (RF_2_, RF_4_, RF_8_, RF_10_) are primarily influenced by the EtOH/H_2_O ratio. This indicates that T mainly enhances mass transfer and the release of compounds from the plant matrix, whereas solvent composition more directly determines the solubility and extraction selectivity of the specific phenolic compounds. The Pareto charts (Figure 1) also aid in visualising that the EtOH/H_2_O ratio and T individually were responsible for 30–73% and 20–40% of the observed changes in various RFs, respectively.

Interestingly, for RF_1_ (yield, g/100 g PM), the EtOH/H_2_O ratio, T, and the quadratic terms are highly influential, indicating strong curvature and temperature-dependent kinetics (Figure 1). Across the models, the quadratic term of the EtOH/H_2_O ratio was frequently the most important non-main effect and was the dominant factor for RF_6_ (TEAC_ABTS_, mg TE/g E), where it showed the highest F-value, indicating that extraction was favoured at intermediate solvent composition rather than at the solvent extremes, consistent with the broad polarity range of phenolic compounds and their differential solubility in ethanol/water mixtures. Consistently high F-values for this term, together with T^2^ in PM-based responses, such as RF_3_ (TPC, mg GAE/g PM), RF_5_ (TEAC_CUPRAC_, mg TE/g PM), and RF_7_ (TEAC_ABTS_, mg TE/g PM), indicate the curvature of the response surface, with extraction plateauing at higher temperatures and achieving maximum recovery of antioxidatively active substances at an intermediate EtOH/H_2_O ratio within the selected PLE operability region.

**Figure 2:**
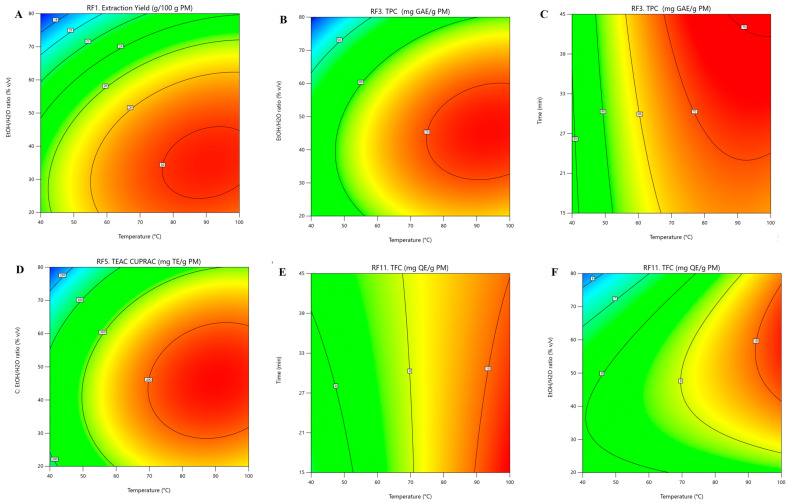
Response surface 2D plots showing the effects PLE-EtOH/H_2_O temperature (T, °C), time (τ, min), and the EtOH/H_2_O ratio (C, % *v*/*v*) and their interactions in *I. salicina*: (**A**) PLE-EtOH/H_2_O extract yield (g/100 g PM); (**B**,**C**) total phenolic content (TPC, mg GAE/g E and PM); (**D**) cupric ion reducing antioxidant capacity (TEAC_CUPRAC_, mg TE/g PM); (**E**,**F**) total flavonoid content (TFC, mg QE/g E and PM).

Overall, extraction τ was the least influential main factor, with low and often non-significant F-values; however, its linear interactions with T and the EtOH/H_2_O ratio were significant for several RFs, indicating that extraction performance depends on the specific combinations of τ with these variables rather than on τ alone (Supplementary Materials, Table S5). The limited influence of τ is consistent with the rapid extraction kinetics of PLE, where elevated T and P promote fast solvent diffusion and solute desorption, so near-equilibrium can be reached within a short time, and prolonged extraction provides little additional recovery [19]. However, its interaction with T and solvent composition indicates that τ may become more relevant under less favourable mass-transfer conditions. The significance of the interaction terms, particularly T × EtOH/H_2_O ratio, further indicates that the effect of temperature depends on solvent composition, reflecting their combined influence on both mass transfer and solvation properties. Specifically, increasing temperature reduces solvent viscosity and surface tension, thereby improving solvent penetration into the plant matrix, while also modifying solvent polarity, which can affect the solubility and solvation capacity of phenolic compounds. The two-dimensional (Figure 2) and the corresponding three-dimensional (Supplementary Materials, Figure S4) contour plots further illustrate the significant interactions among T, τ, and the EtOH/H_2_O ratio for the selected PM-based responses (RF1, RF3, RF5 and RF11).

For extraction yield (RF_1_; Figure 2A), the surface as a function of T and the EtOH/H_2_O ratio shows strong curvature, with closed elliptical contours, confirming a significant interaction between these variables within the PLE operating region. Yield increases sharply as T increases from 40 to about 80–90 °C, especially when the solvent changes from ethanol-rich (>70%) to more aqueous mixtures (30–50%), with a maximum at 80–90 °C and an EtOH concentration of 30–40%. At higher EtOH content (>60%), a decrease in extraction yield is observed, despite the elevated T. The response surfaces for TPC (RF_3_; Figure 2B) and TEAC_CUPRAC_ (RF_3_; Figure 2D) exhibit a dome-shaped profile similar to RF_1_, with nearly concentric contour lines that indicate a strong interaction between T and EtOH/H_2_O ratio across the studied domain. The highest values are obtained at the upper part of the T range (>85 °C) combined with an intermediate EtOH/H_2_O ratio (30–50%), with lower responses in both ethanol-rich (>70%) and water-rich (<30%) media, confirming that the intermediate polarity of hydroethanolic mixtures is critical for efficient antioxidatively active phenolic compound recovery from *I. salicina*. When TPC is plotted as a function of T and τ (Figure 2C), the relatively flat response along the τ axis compared with T indicates that the equilibrium of the PLE extraction, which is mainly governed by T, is reached rapidly, and that extending τ beyond 30 min yields only negligible additional TPC. Similarly, for TFC (RF_11_), the T–τ surface (Figure 2E) is almost planar, with nearly parallel contours, indicating an approximately linear positive effect of T and only a minimal influence of τ on this RF. In contrast, the T-EtOH/H_2_O surface (Figure 2F) shows that the TFC values increase with T (>80 °C) when the EtOH concentration in the solvent is at 50–70%; however, they remain low at EtOH < 40%, even at elevated T, suggesting that the polarity of the hydroethanolic solvent, rather than heat-induced mass transfer, is the main factor controlling flavonoid recovery from *I. salicina* during PLE.

Considering these observations, multi-response optimisation using a desirability function was applied to identify the optimal PLE EtOH/H_2_O conditions. The optimisation constraints were set to obtain high-yield extracts (>28 g/100 g PM) while minimising T and τ, maintaining the EtOH/H_2_O ratio within 20–80% (*v*/*v*), and ensuring that all PM-based response factors (RFs) remained > 90% of their respective maximum values within the selected PLE operating region. The corresponding thresholds were: TPC > 65 mg GAE/g PM, TEAC_CUPRAC_ > 378 mg TE/g PM, TEAC_ABTS_ > 238 mg TE/g PM, TEAC_ORAC_ > 304 mg TE/g PM, and TFC > 9.5 mg QE/g PM. As indicated by Design-Expert software, these objectives can be met by performing PLE at 82–95 °C for ≥27 min using a hydroethanolic solvent with an EtOH concentration of 55–65% (*v*/*v*), achieving an overall desirability >0.57 and highlighting the complexity of multi-response optimisation when the optima for individual RFs occur in different regions of the experimental space (Figure 2). For instance, extraction at 82 °C for 27 min with a 60:40 (%, *v*/*v*) EtOH/H_2_O mixture yielded approximately 29 g/100 g PM of polar extract, corresponding to 66 mg GAE of TPC and nearly 10 mg QE of TFC recovery per gram of PM, as well as high in vitro antioxidant capacity values (TEAC_CUPRAC_ 426 mg TE/g PM, TEAC_ABTS_ 253 mg TE/g PM, TEAC_ORAC_ 339 mg TE/g PM) (Table 3). The close agreement between the experimental and predicted values under the identified optimal conditions further confirmed the validity of the models (Supplementary Materials, Table S7). In addition, TPC and the in vitro antioxidant activity (TEAC_CUPRAC_ and TEAC_ABTS_) values of the residual PM after PLE-EtOH/H_2_O were markedly (>92%) lower than those of *I. salicina* before extraction, indicating that the majority of antioxidant-active constituents were efficiently recovered by applying optimised PLE conditions (Table 3).

To the best of our knowledge, this is the first report describing the use and optimisation of PLE to obtain antioxidant-rich fractions at high yields from plants of the genus *Inula.* Generally, published applications of other intensified extraction technologies to obtain valuable constituents from these medicinal plants remain scarce. For example, previously, ultrasound-assisted extraction (UAE) was employed to isolate phenolic compounds [29], flavonoids [30] and the sesquiterpene lactones alantolactone and isoalantolactone [25] from *I. helenium* roots. Recently, the efficiency of UAE versus the conventional Soxhlet extraction was demonstrated for *I. viscosa* as well, under optimised conditions (54% EtOH, 60 °C, 31 min, solvent-to-solid ratio 15 mL/g), yielding approximately 25 g/100 g of polar fraction, with high TPC (122 mg GAE/g PM) and TFC (37 mg QE/g PM) values [31]. Similarly, there are only a few published reports on the optimisation of subcritical water and microwave-assisted extractions, as well as high-pressure homogenisation, for isolating sesquiterpene lactones and flavonoids from *I. racemose* aerial parts [32] and *I. helenium* roots [26,27,28].

### 3.3. Validation of CCD-RSM Optimisation Using Aspen Plus^®^ Simulation

**Figure 3:**
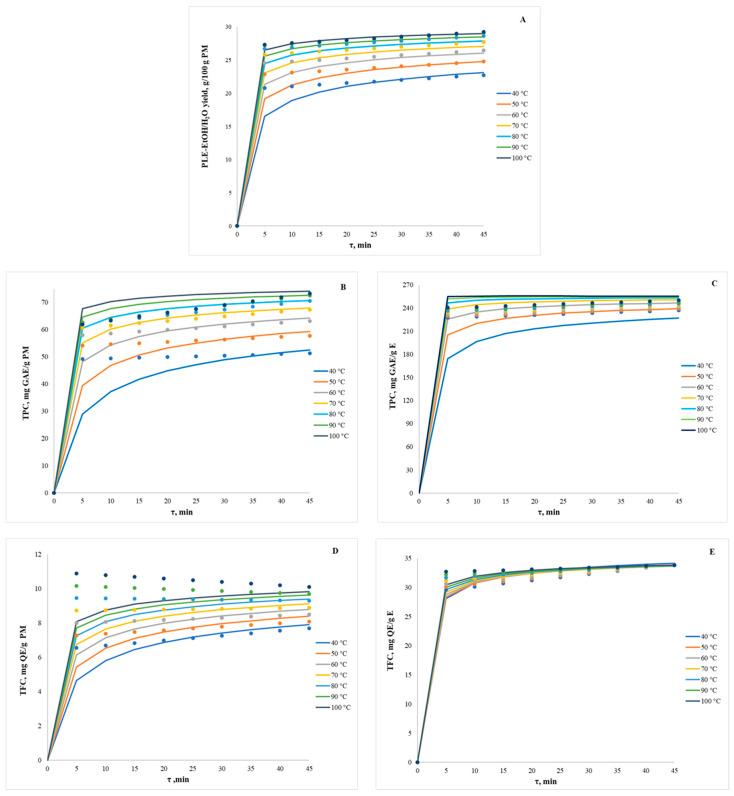
Comparison of CCD-RSM (dots) and Aspen Plus^®^ (lines) modelling of *I. salicina*: (**A**) PLE- EtOH/H_2_O extract yield (g/100 g PM); (**B**,**C**) total phenolic content (TPC, mg GAE/g PM and E); (**D**,**E**) total flavonoid content (TFC, mg QE/g PM and E).

To simulate the PLE process in Aspen Plus^®^, the complex phytochemical composition of *I. salicina* was represented by a set of selected reference compounds, namely gallic acid, quercetin, and Trolox, which were used to represent the major phenolic classes (phenolic acids and flavonoids) and the overall fraction of polar antioxidatively active constituents. Therefore, first- and second-order polynomial regression equations in actual factors for RF_1_ (PLE-EtOH/H_2_O yield, g/100 g PM), RF_2_ and RF_3_ (TPC, mg GAE/g E and PM), and RF_10_ and RF11 (TFC, mg QE/g E and PM) (Table S6, Supplementary Materials), obtained from the mathematical modelling, were used to generate input data for the Aspen Plus^®^ rate-based extractor model (Figure 3). Although this approach does not fully capture the complexity of the plant matrix, including matrix–solute effects and possible interactions among phytochemicals during extraction, it provides a simplified and comparable simulation model that can describe the general extraction behaviour of the main target phytochemical groups in *I. salicina* and support industrially relevant extraction process interpretation.

For RF_1_ (Figure 3A), the CCD-RSM and Aspen Plus^®^ models overlap closely across the experimental T range, indicating that the overall yield is governed by reproducible thermodynamic and mass-transfer behaviour, which the physical model represents well. Across all conditions, most of the extract is obtained during the first 10 min of extraction, after which the yield increases only marginally with prolonged τ. Raising the T from 40 to 100 °C increases the final yield by about 50%. The steepness of the kinetic curves indicates an apparent extraction order >1, consistent with rapid extraction of readily accessible solutes, followed by slower diffusion-controlled release from the plant matrix. The relatively small additional yield increase at higher T further suggests a relatively low apparent activation energy. TPC recovery per gram of PM (Figure 3B) closely follows the overall yield, with the CCD-RSM and Aspen Plus^®^ models in good agreement, and increases with both τ and T, consistent with higher phenolic solubility and diffusivity and enhanced desorption at elevated T. Small deviations between both models may be observed at lower T (<60 °C) and intermediate τ (0–20 min), however, indicating the same plateauing trend and the overall T dependence. When TPC is expressed on an E basis (Figure 3C), the T effects are generally weak, and the curves converge after the initial period of extraction, with Aspen Plus and CCD-RSM models nearly overlapping. This indicates that a higher T primarily increases extract recovery (and thus TPC per gram of PM) rather than enriching phenolics in the extract, implying near-proportional co-extraction with the bulk soluble fraction. Therefore, the process activation energy, order, and kinetic factor of TPC should be similar to the formation of the PLE-EtOH/H_2_O yield. However, the RF_10_ and RF_11_ plots (Figure 3D,E) show the most apparent model-dependent differences. For example, CCD-RSM data suggest that TFC per PM basis values (Figure 3D) slightly increase with τ and T < 70 °C; however, at higher T, it declines with prolonged τ, and Aspen Plus^®^, in contrast, predicts a smooth progression towards a plateau throughout the entire experimental T range. These differences suggest that additional mechanisms may influence TFC under elevated T and prolonged τ (e.g., thermal or oxidative loss of labile flavonoids, ring-opening reactions or matrix interactions that reduce the QE-equivalent signal despite enhanced mass transfer), which are captured by CCD-RSM but are not well represented in the standard Aspen mass-transfer/equilibrium framework without adding explicit degradation or multi-component reactions. These discrepancies may also arise from differences in solubility among flavonoid subclasses, which are related to their polarity and chemical structure. For example, highly alkylated aglycones are better extracted with less polar solvents, whereas hydroxylated aglycones and especially glycosylated flavonoids are more efficiently extracted with alcohol, water, or their mixtures; moreover, glycosylation enhances solubility [57]. In contrast, when expressed on an E basis (Figure 3E), the TFC curves overlap strongly across all T after the first minutes of extraction, indicating that flavonoids are extracted predominantly at the beginning of PLE regardless of T, while subsequent extraction mainly increases co-extracted non-flavonoid material and therefore dilutes TFC in the extract. From a practical perspective, this behaviour suggests a negligible apparent activation energy for flavonoid extraction and indicates that elevated T and extended τ would provide little additional benefit if maximising flavonoid content were the primary objective of the PLE optimisation. Aspen Plus^®^ calculations indicate that the discussed RFs are optimised at moderate PLE conditions, approximately 80 °C and 25 min, since higher T and τ provide negligible additional increases in values, which is in close agreement with the CCD-RSM-derived optimal conditions.

The volume of the extraction zone for 1 kg/h of PM and 21.04 kg/h of EtOH/H_2_O ranges from 19.05 to 20.48 litres, depending on the T of PLE; therefore, a 25-litre extractor would be sufficient for this extraction process. Falling-film evaporation was selected as the most suitable method for removing the EtOH/H_2_O mixture after extraction, under vacuum, providing efficient heat and mass transfer with minimal thermal degradation of heat-labile compounds. In a falling-film evaporator, the liquid forms a thin film that flows along the heated surface, allowing rapid solvent evaporation at relatively low temperatures due to high heat transfer coefficients and short residence times. This is especially important for EtOH/H_2_O solvent systems, where precise T control helps achieve selective EtOH evaporation while minimising energy consumption. In Aspen Plus^®^, the falling-film evaporation process is typically modelled using one or a series of flash evaporators to approximate vapour–liquid equilibrium behaviour at different temperature and pressure stages. This approach enables the steady-state simulation of solvent removal while capturing the phase separation and concentration changes at each step, consistent with the thermodynamic behaviour of the EtOH/H_2_O solvent system. For this study, a single flash evaporator operated at the extraction T was used to model EtOH/H_2_O removal, and vacuum levels of 10–200 mbar with T up to 65 °C were evaluated to minimise thermal degradation of heat-labile compounds. The data in Figure S5 (Supplementary Materials) illustrate how the solvent mass fraction in the PLE-EtOH/H_2_O extract depends on both T (Figure S5A) and P (Figure S5B), outlining the coupled effects of these parameters on the falling-film evaporation efficiency. For example, at higher P (150–200 mbar), the solvent content remains high across the entire T range (45–65 °C), indicating limited evaporation (Figure S5A). As P decreases, especially to <100 mbar, the solvent fraction declines sharply with increasing T, showing that vacuum operation significantly enhances evaporation even at moderate T. At 10 mbar, nearly complete solvent removal is achieved above 45 °C, demonstrating that low-pressure conditions are the most effective for solvent stripping. The data in Figure S5B confirm this behaviour: for each fixed T, the solvent fraction rises steeply with increasing P, especially above 100 mbar. At lower T (45–50 °C), evaporation is highly pressure-sensitive, while at higher T (60–65 °C), the curves flatten, indicating that T becomes the dominant factor and P influence weakens. The modelling results show that optimal evaporation of EtOH/H_2_O can be achieved by operating the flash evaporator at P < 100 mbar and T of 60–65 °C, balancing effective solvent removal with minimal thermal degradation of the extract’s bioactive compounds. Evaporator energy consumption depends on the extraction conditions and the required evaporation efficiency; even assuming that extraction proceeds at an optimal 80 °C, the evaporator still needs an external heat supply, as only a small fraction of EtOH/H_2_O can be removed just by reducing pressure and applying vacuum. Evaporator duty strongly depends on the amount of EtOH/H_2_O removed and requires approximately 8.6 kW of power to achieve the desired extract purity at P < 100 mbar (Figure S6, Supplementary Materials).

Other studies have likewise highlighted the value of combining mathematical modelling with physical process simulation to optimise PLE conditions for the recovery of bioactive constituents. For example, Ferro et al. reported the successful scale-up of PLE for *Sida rhombifolia* leaves by combining Sovová’s model with a MatLab R2016a-based simulation extraction model, using small-scale kinetic data to predict pilot- and large-scale extraction performance, and confirming scale equivalence by comparing yield, TPC, and antioxidant activity of extracts measured by the DPPH^•^ and ABTS^•+^-scavenging assays [58].

### 3.4. Phytochemical Profile, In Vitro Antioxidant and Photoprotective Properties of I. salicina PLE-EtOH/H_2_O Extract Under Optimised Extraction Conditions

In the following part of this study, the phytochemical profile of the *I. salicina* extract obtained under optimal PLE-EtOH/H_2_O conditions (82 °C, 27 min, EtOH/H_2_O 60/40% *v*/*v*) was assessed using UPLC-ESI/MS^2^. In total, 40 compounds were tentatively identified based on their parental and fragment ions and patterns, retention order, and comparison with available literature data (Table 4).

The extract was primarily characterised by the presence of chlorogenic acid (CGA) and its dicaffeoyl derivatives, mainly, 3,5-, 1,5-, 4,5-, and 3,4-dicaffeoyl esters. The results of the quantitative analysis of these major phenolic acids are reported in Table 5. As shown, the main constituent was CGA, with a content of 96.9 mg/g E, corresponding to ~28.2 mg/g PM. This observation and reported content are in close agreement with a few available reports on the phytochemical composition of *I. salicina* extracts. Specifically, Ivanova et al. reported that CGA was the most abundant phenolic substance in *I. salicina* methanolic extract, with CGA accounting for a total of 103.4 mg/g E [12]. Moreover, Péter and Dósa analysed five Hungarian *Inula* species and quantified three major components: CGA, caffeic acid, and hyperoside [59]. Among these, *I. salicina* had the highest CGA content, although its levels were significantly lower than those reported in the present study. Additionally, CGA has been previously identified as a significant component of the flowers of six Bulgarian *Inula* species, distinct from those of this study, with its content ranging from 13.0 to 28.4 mg/g in *I. aschersoniana* and *I. ensifolia*, respectively [60]. It is worth noting that CGA is known for its antioxidant and anti-inflammatory properties, which contribute to its therapeutic potential for managing conditions such as diabetes, cardiovascular and neurodegenerative diseases, cancer, and hypertension [61]. It also supports gut health and exerts its effects through mechanisms such as free radical scavenging, cytokine regulation, and modulation of metabolic pathways. Besides CGA, 4 di-caffeoyl isomers of CGA were characterised in the *I. salicina* PLE extract obtained under optimal conditions, with their contents presented in Table 3. Two dicaffeoyl esters, specifically the 3,5- and 1,5 dicaffeoylquinic acids, were present in high amounts, contributing ~65 mg/g E each (Table 5). The other two isomers, 3,4- and 4,5-dicaffeoylquinic acids, were determined to have lower amounts, contributing 6.5 and 18.4 mg/g E, respectively (Table 5). From a pharmacological standpoint, these compounds exhibit a wide range of biological properties, including antioxidant, cardiovascular-protective, antibacterial, antiviral, blood sugar-lowering, and neuroprotective properties, and have shown potential for treating respiratory diseases [62]. Moreover, Wu et al. reported that 1,3-O-dicaffeoylquinic acid was among the monomeric substances identified in *I. capppa*, which may influence signal transduction pathways, including the TLR2/MyD88/NF-κB pathway involved in anti-inflammatory responses [63]. Previous reports have also identified dicaffeoylquinic acids as major compounds of *I. salicina*, but in smaller quantities than in this study, ranging from 5.4 to 30.6 mg/g E, and increasing in the order 3,4-< 1,5- < 4,5- < 3,5-dicaffeoylquinic acid [12]. Another study also indicated that 1,5-dicaffeoyl quinic acid was the dominant isomer, with its content ranging across different *Inula* species from 5.5 to 28.4 mg/g E [60]. Additionally, as reported in Table 4, various other substances were assigned to different phenolic classes based on their parental and fragment ions, as well as previous reports in the literature for species of this genus. Besides the aglycons and glycosylated forms of hydroxycinnamic and hydroxybenzoic acids, acylquinic acids, caffeoylhexaric acids, and glycosylated flavonoids such as isoquercetin and kaempferol glucoside were also detected.

The high levels of CGA and its derivatives (Table 5), together with other tentatively identified phenolics (Table 4), account for the elevated TPC (227 mg GAE/g E) and TEAC values of the *I. salicina* PLE-EtOH/H_2_O extract, measured by ABTS (869 mg TE/g E), CUPRAC (1473 mg TE/g E), and ORAC (1165 mg TE/g E) assays (Table 3). Overall, the reported antioxidant capacity in plants of the genus *Inula* varies widely across species, geographic origin, extraction conditions, differences in phytochemical composition, and other factors. For example, recently, Ceylan et al. reported that methanolic extracts from the aerial parts of Turkish *Inula* species contained 47–100 mg GAE/g of TPC and 31–71 mg rutin equivalents/g of TFC, with substantial variability in radical-scavenging activity (DPPH: 60–188 mg TE/g; ABTS: 91–221 mg TE/g) and reducing power (CUPRAC: 170–461 mg TE/g; FRAP: 82–238 mg TE/g). These differences were attributed to species-dependent phytochemical profiles, with quinic acid (9–42 mg/g) and chlorogenic acid (7–24 mg/g) as major constituents in eight of eleven species, including *I. viscidula* and *I. mariae*, which exhibited the highest antioxidant capacity in these assays. The particularly high activity of *I. viscidula* extract was further associated with its elevated astragalin content (~20 mg/g E). Notably, rutin (0.1–79 mg/g) was the predominant metabolite in *I. discoidea* and *I. aucheriana* and was mainly associated with metal-chelating activity and acetylcholinesterase inhibition rather than radical-scavenging capacity, consistent with its reported tyrosinase- and α-glucosidase-inhibitory effects [49]. In another study, ethanolic maceration of *I. viscosa* aerial parts collected in Northeast Algeria yielded extracts with comparably high TPC (145 mg GAE/g E) and TFC (22 mg QE/g E) [64]; however, the reported TPC values for *I. viscosa* vary widely (75–299 mg GAE/g E) due to differences in solvent composition, extraction conditions, geographic origin, and harvest season [50,65,66,67]. For example, Brahmi-Chendouh et al. fractionated a Soxhlet methanolic extract of *I. viscosa* leaves collected in Algeria and obtained an ethyl acetate fraction with a TPC of 299 mg GAE/g E and strong dose-dependent antioxidant activity (87% DPPH^•^ and ~68% ABTS^•+^-scavenging at 50 mg/mL), attributing the antioxidant activity mainly to caffeoyl shikimic acids and dihydrobenzofuran lignans [50]. In another study, secondary metabolite profiles and antioxidant capacity of *I. helenium* ethanolic Soxhlet extracts varied by anatomical part, sampling time, and site: TPC was highest in inflorescences (90 mg GA/g), TFC peaked in leaves (376 mg rutin equivalents/g), and the DPPH^•^-scavenging capacity was detected in roots (IC_50_ of 162 µg/mL), consistent with their higher phenolic acid content (e.g., caffeic acid); also, antioxidant activity was higher in plants collected at higher altitudes during summer months than in those from lower-altitude sites or sampled in autumn [52].

In addition to antioxidant activity, high contents of hydroxycinnamic acids and their derivatives support the potential use of *I. salicina* PLE-EtOH/H_2_O extract in skincare applications due to the photoprotective effects of these phytochemicals [68,69]. Exposure to sunlight is known to cause cumulative dermal damage, leading to skin ageing and an increased risk of skin cancer. For these reasons, various studies emphasise the importance of consistent use of sunscreen containing antioxidant components, which offer additional protection against UV rays, infrared-A (IRA), pollution-induced oxidative stress, and anti-ageing benefits [70,71]. In the European Union, the efficacy and safety of sunscreen products are regulated under Cosmetic Products Regulation (EC) No. 1223/2009, which defines the UV filters permitted for cosmetic use and their maximum allowed concentrations in ready-to-use formulations, typically ranging from 2% to 25% (*w*/*v*). Among these, titanium dioxide (TiO_2_) is a widely used inorganic UV filter that provides broad-spectrum UV-A/UV-B protection and, at 25% (*w*/*v*), has been reported to achieve an in vitro sun protection factor (SPF) of ~38 [72]. In practice, sunscreen formulations commonly combine multiple UV filters, as synergistic interactions among active compounds are often necessary to achieve higher SPF values and broader spectral coverage. For example, combinations of TiO_2_ with synthetic organic UV filters, such as phenylbenzimidazole sulfonic acid, octyl methoxycinnamate, and isoamyl p-methoxycinnamate, have been reported to increase SPF values to ~50 [73]. However, the photoprotective efficacy of TiO_2_ depends strongly on particle size and formulation properties, while its use remains under evaluation because of potential health and environmental risks, including oxidative stress, possible genotoxicity, and UV-induced photocatalytic damage to skin components [72]. As the effectiveness and safety of many synthetic sunscreen agents are limited by photoinstability, toxicity, and environmental impact, increasing attention has been directed towards plant-derived alternatives. Medicinal plants have evolved efficient defence mechanisms against UV-induced oxidative stress, such as the hydroxycinnamic acid derivatives, and many reviews highlight the potential of these natural antioxidants as eco-friendly sun-protective alternatives [68,69,74,75]. A well-known example is the standardised aqueous leaf extract of *Polypodium leucotomos* (marketed as Fernblock^®^), which contains 0.6–1.3% total phenolic compounds and 0.4–0.9% quinic acid [76]. Used both topically and orally, this extract has shown photoprotective activity against UV-induced damage and has been applied clinically in photodermatoses, pigmentary disorders, and as an adjunct to photodynamic therapy for actinic keratoses [77]. Interestingly, Salazar-Chacón et al. recently reported that a hydromethanolic leaf extract of wild *P. leucotomos* exhibited an in vitro SPF of 20, placing it in the mid-range among 19 evaluated fern species, whereas higher SPF values (~30) were obtained for *Pecluma consimilis*, *Serpocaulon sessilifolium*, and *Serpocaulon dissimile* extracts [78].

The in vitro photoprotective activity of the *I. salicina* PLE-EtOH/H_2_O extract under optimised conditions was assessed at different concentrations by calculating SPF using the Mansur method, which estimates UV-B protection from weighted absorbance in the 290–320 nm range, where erythemally effective UV-B radiation is highest [79]. As reported in Table S8 (Supplementary Materials), SPF increased with extract concentration from 0.86 at 5 µg/mL to ~50 at 1.0 mg/mL. An SPF of 10 was achieved at approximately 58 µg/mL, in close agreement with the only other reported SPF for an *Inula* species, where Ivanova et al. reported an SPF of 10 for a methanolic *I. salicina* extract at 62.5 µg/mL [12]. According to the Commission Recommendation on the efficacy of sunscreen products and the claims made in relation to them (2006/647/EC), sunscreen efficacy is classified as low (SPF 6–10), medium (SPF 15–25), high (SPF 30–50), and very high (SPF ≥ 50). Within this framework, the *I. salicina* PLE-EtOH/H_2_O extract is in the upper “high” range already at concentrations >0.25 mg/mL (SPF > 40) and, at >0.5 mg/mL, approaches the “very high” threshold (~50), corresponding to >97% UV-B absorption potential (Supplementary Materials, Table S8). Interestingly, when CGA is evaluated at concentrations comparable to its level in the *I. salicina* PLE-EtOH/H_2_O extract (approximately ~10% of the extract mass, Table 3), an SPF of 10 is reached at ~20 µg/mL, consistent with previous observations [74]. However, the extract provides comparable protection at substantially lower CGA-equivalent concentrations, indicating that the photoprotective response cannot be attributed solely to CGA, and that other constituents contribute additively and/or synergistically to UV-B screening. A plausible explanation is that co-extracted phenolics (including other hydroxycinnamates and flavonoid-type compounds) provide overlapping absorption bands in the UV-B region and may also stabilise each other against photo-oxidation, thereby enhancing the net absorbance captured by the Mansur calculation. Moreover, estimated CGA contribution to SPF value increases with extract concentration (25–30% at ≤0.25 mg/mL, ~50% at 0.5 mg/mL, and up to ~82% at 1 mg/mL; Supplementary Materials, Table S8), suggesting that CGA becomes increasingly dominant at higher loadings, while the broader phenolic content strongly influences the remaining protection at lower concentrations. Although direct synergy between CGA and other phenolics has not been explicitly demonstrated to enhance photoprotective activity, studies suggest that various phenolic compounds in complex mixtures, such as plant extracts, may act synergistically, thereby increasing overall biological activity [80].

## 4. Conclusions

*I. salicina* represents a promising renewable feedstock for obtaining phenolic-rich extracts with multiple potential applications across functional product industries, and optimised PLE-EtOH/H_2_O is highly effective for recovering high-value-added antioxidant fractions from this complex plant matrix. By integrating multi-response CCD-RSM with Aspen Plus^®^ process modelling, optimal PLE conditions were identified (82 °C, 27 min, 60% EtOH *v*/*v*), yielding ~29 g extract per 100 g PM, characterised by high TPC (227 mg GAE/g E), TFC (34 mg QE/g E), and TEAC values in CUPRAC, ABTS^•+^-scavenging and ORAC assays (869–1473 mg TE/g E). TPC and TEAC values of the post-extraction plant residue were by >92% lower than those of the unextracted *I. salicina*, confirming that the majority of antioxidant-active constituents were efficiently extracted with PLE-EtOH/H_2_O. Compared with conventional Soxhlet extraction, PLE increased phenolic and antioxidant recovery by up to two-fold, while reducing extraction time from 6 h to <30 min. Phytochemical profiling by UPLC-ESI/MS^2^ demonstrated that the *I. salicina* extract was dominated by hydroxycinnamate derivatives, with chlorogenic acid as the major marker compound (~97 mg/g E), accompanied by several dicaffeoylquinic acid isomers (notably 3,5-, 1,5-, 4,5-, and 3,4-dicaffeoylquinic acids) as prominent co-constituents. Collectively, chlorogenic acid and these caffeoylquinic acid derivatives accounted for a substantial fraction of the extract mass (~25%), consistent with the extract’s high antioxidant capacity and marked photoprotective potential (SPF ~50 at 0.5 mg/mL). The Aspen Plus^®^ simulations aligned with the CCD-RSM data and supported the technical feasibility of scale-up, including efficient downstream solvent removal via falling-film evaporation under reduced pressure (<100 mbar). This approach supports green chemistry and circular bioeconomy principles by improving resource efficiency and plant biomass utilisation through extraction intensification, while minimising environmental impacts through optimised operating conditions (particularly temperature and extraction time).

To the best of our knowledge, this study provides one of the first integrated experimental and process-modelling approaches, demonstrating the potential of an optimised and scalable PLE process to produce higher value-added *I. salicina* fractions suitable for pharmaceutical, nutraceutical, and cosmetic applications. Nevertheless, future research should expand the functional characterisation of both crude and fractionated extracts (e.g., antimicrobial, anti-inflammatory, physiologically important enzyme-inhibiting properties, etc.) while linking these outcomes to detailed phytochemical profiling. In parallel, scale-oriented techno-economic and life-cycle assessments could be performed by incorporating solvent recycling, energy demand, and downstream concentration and purification steps, along with stability and batch-to-batch variability studies, to strengthen industrial implementation and regulatory readiness.

## Figures and Tables

**Table 1:** Yields, total phenolic content (TPC), in vitro cupric ion reducing antioxidant capacity (TEAC_CUPRAC_) and ABTS^•+^-scavenging capacity (TEAC_ABTS_) of polar *I. salicina* extracts obtained by Soxhlet and pressurised liquid extractions.

Properties	Units	*I. salicina* Extracts				
		SE-EtOH	PLE-EtOH	PLE-EtOH/H_2_O-1	PLE-EtOH/H_2_O-2	PLE-H_2_O
Yield	g/100 g PM	10.81 ± 0.53 ^a^	11.61 ± 0.06 ^a^	27.70 ± 1.70 ^b^	29.24 ± 0.67 ^b^	35.36 ± 3.78 ^c^
TPC	mg GAE/g E	139.65 ± 2.62 ^a^	263.43 ± 16.86 ^c^	258.17 ± 3.32 ^c^	251.66 ± 6.71 ^c^	228.55 ± 3.02 ^b^
	mg GAE/g PM	15.10 ± 0.28 ^a^	30.58 ± 1.96 ^b^	71.51 ± 0.92 ^c^	73.58 ± 1.96 ^c^	80.82 ± 1.07 ^d^
In vitro antioxidant activity:						
TEAC_CUPRAC_	mg TE/g E	1149.79 ± 94.46 ^a^	1555.02 ± 34.59 ^c^	1418.02 ± 110.27 ^bc^	1332.33 ± 117.54 ^b^	1338.11 ± 84.77 ^b^
	mg TE/g PM	124.59 ± 10.21 ^a^	180.54 ± 4.02 ^b^	392.79 ± 30.55 ^c^	389.57 ± 34.37 ^c^	473.16 ± 29.97 ^d^
TEAC_ABTS_	mg TE/g E	517.97 ± 25.11 ^a^	951.89 ± 35.25 ^d^	907.63 ± 21.81 ^cd^	874.76 ± 22.01 ^c^	823.10 ± 27.04 ^b^
	mg TE/g PM	55.99 ± 2.71 ^a^	110.51 ± 4.09 ^b^	251.41 ± 6.04 ^c^	255.78 ± 6.44 ^c^	250.77 ± 24.17 ^c^

PM: plant material (*I. salicina*); TPC: total phenolic content; CUPRAC: cupric ion reducing antioxidant capacity; ABTS^•+^-scavenging capacity; E: extract; GAE: gallic acid equivalents; TE: Trolox equivalents; TEAC: Trolox equivalent antioxidant capacity. PLE-EtOH: pressurised ethanol extraction (45 min, 70 °C, 10.3 MPa); PLE-EtOH/H_2_O-1: pressurised ethanol/water extraction (45 min, 70 °C, 10.3 MPa, EtOH/H_2_O: 70/30% *v*/*v*); PLE-EtOH/H_2_O-2: pressurised ethanol/water extraction (45 min, 70 °C, 10.3 MPa, EtOH/H_2_O: 50/50% *v*/*v*); PLE-H_2_O: pressurised water extraction (45 min, 110 °C, 10.3 MPa); SE-EtOH: Soxhlet extraction with ethanol (6 h, 78 °C). Extraction yields are reported as the mean of three technical replicates ± SD; TPC and TEAC values are reported as the mean of four technical replicates ± SD. The different superscript letters in the same line indicate significantly different values (*p* < 0.05; based on a one-way ANOVA).

**Table 2:** Central composite design matrix and observed response values (per gram of plant material) for PLE-EtOH/H_2_O extraction of polar constituents from *I. salicina*.

CCD	PLE-EtOH/H_2_O Parameters			RF_1_ Yield	RF_3_ TPC	RF_5_ TEAC_CUPRAC_	RF_7_ TEAC_ABTS_	RF_9_ TEAC_ORAC_	RF_11_ TFC
Run No.	T, °C	τ, min	EtOH/H_2_O, % *v*/*v*	g/100 g PM	mg GAE/g PM	mg TE/g PM	mg TE/g PM	mg TE/g PM	mg QE/g PM
1	70	30	50/50	27.63 ± 1.03	65.35 ± 0.85	400.24 ± 36.83	253.16 ± 10.84	305.63 ± 21.33	9.01 ± 0.14
2	40	15	20/80	25.32 ± 1.50	52.17 ± 0.72	293.92 ± 7.10	164.04 ± 14.28	260.20 ± 8.98	7.25 ± 0.01
3	70	30	50/50	29.02 ± 0.35	69.56 ± 1.04	393.97 ± 10.21	265.10 ± 2.15	314.93 ± 7.06	8.66 ± 0.14
4	70	30	50/50	28.77 ± 0.35	71.05 ± 1.27	415.55 ± 7.49	258.56 ± 13.14	318.93 ± 9.62	9.14 ± 0.24
5	70	30	20/80	28.27 ± 0.24	62.04 ± 2.64	353.54 ± 6.52	215.85 ± 5.40	316.91 ± 9.83	8.15 ± 0.18
6	70	30	50/50	29.15 ± 0.35	72.59 ± 1.73	420.49 ± 16.72	261.48 ± 13.18	336.13 ± 6.52	9.46 ± 0.23
7	70	30	50/50	28.84 ± 0.35	66.81 ± 1.15	384.53 ± 9.12	248.45 ± 8.74	337.79 ± 8.40	9.44 ± 0.10
8	100	30	50/50	30.30 ± 1.73	71.28 ± 3.03	412.95 ± 10.89	265.83 ± 6.77	338.80 ± 14.35	9.41 ± 0.16
9	70	15	50/50	27.64 ± 0.28	64.50 ± 1.09	366.17 ± 5.08	233.01 ± 6.03	313.43 ± 26.53	8.74 ± 0.20
10	40	15	80/20	15.92 ± 0.83	38.17 ± 0.82	211.14 ± 7.25	127.74 ± 8.34	181.80 ± 3.31	4.96 ± 0.16
11	70	30	50/50	28.24 ± 0.35	67.68 ± 1.18	406.56 ± 10.79	243.97 ± 16.47	308.37 ± 23.54	8.93 ± 0.23
12	70	30	80/20	22.98 ± 1.21	56.14 ± 0.99	326.36 ± 4.31	194.09 ± 11.16	260.78 ± 4.38	8.17 ± 0.10
13	100	45	80/20	24.46 ± 0.47	65.89 ± 0.56	377.13 ± 3.68	200.13 ± 4.00	275.02 ± 10.03	9.09 ± 0.39
14	40	30	50/50	23.49 ± 1.59	54.27 ± 0.53	292.25 ± 7.33	197.33 ± 8.23	259.18 ± 14.81	7.66 ± 0.11
15	100	15	80/20	22.85 ± 0.24	55.02 ± 0.72	318.21 ± 11.15	186.26 ± 9.33	263.98 ± 4.70	10.57 ± 0.17
16	100	45	20/80	30.79 ± 0.45	68.60 ± 0.64	387.45 ± 2.36	238.20 ± 8.98	317.03 ± 25.49	8.49 ± 0.09
17	40	45	80/20	16.21 ± 0.09	39.44 ± 0.17	227.95 ± 11.47	150.29 ± 5.61	195.50 ± 7.03	5.73 ± 0.18
18	40	45	20/80	26.58 ± 0.35	54.03 ± 1.23	298.77 ± 26.89	207.22 ± 4.59	261.26 ± 5.44	7.79 ± 0.13
19	100	15	20/80	28.36 ± 0.49	62.41 ± 1.92	359.02 ± 2.53	229.95 ± 7.50	323.25 ± 15.73	8.59 ± 0.19
20	70	45	50/50	29.24 ± 0.05	69.54 ± 0.73	408.37 ± 4.96	260.86 ± 8.94	328.41 ± 9.47	9.65 ± 0.28

EtOH/H_2_O: pressurised ethanol/water extraction; PM: plant material (*I. salicina*); TPC: total phenolic content; GAE: gallic acid equivalents; CUPRAC: cupric ion reducing antioxidant capacity; ORAC: oxygen radical absorbance capacity; QE: quercetin equivalents; RF: response factor; TFC: total flavonoid content; TE: Trolox equivalents; TEAC: Trolox equivalent antioxidant capacity. PLE-EtOH/H_2_O yields are reported as the mean of three technical replicates ± SD; TPC, TFC, and TEAC values are reported as the mean of four technical replicates ± SD.

**Table 3:** Yields, total phenolic (TPC), flavonoid (TFC) content, in vitro antioxidant activity (TEAC_CUPRAC_, TEAC_ABTS_, TEAC_ORAC_), and selected phenolic acid content of *I. salicina* polar extract and plant material before and after PLE-EtOH/H_2_O extraction under optimal conditions (82 °C, 27 min, EtOH/H_2_O 60/40% *v*/*v*).

Properties	Units	*I. salicina* Samples		
		PLE-EtOH/H_2_O_OPT_ Extract	PM Prior PLE-EtOH/H_2_O_OPT_	PM After PLE-EtOH/H_2_O_OPT_
Yield	g/100 g PM	29.10 ± 0.46	-^na^	-^na^
TPC	mg GAE/g E	227.39 ± 3.45		
	mg GAE/g PM	66.17 ± 1.00 ^b^	82.52 ± 6.69 ^c^	6.23 ± 0.58 ^a^
TFC	mg QE/g E	34.12 ± 1.15		
	mg QE/g PM	9.93 ± 0.34	-^na^	-^na^
In vitro antioxidant activity:				
TEAC_CUPRAC_	mg TE/g E	1472.91 ± 52.44		
	mg TE/g PM	428.62 ± 15.26 ^b^	499.60 ± 47.22 ^c^	15.82 ± 0.72 ^a^
TEAC_ABTS_	mg TE/g E	868.73 ± 47.19		
	mg TE/g PM	252.80 ± 13.73 ^b^	268.64 ± 20.18 ^b^	10.87 ± 0.91 ^a^
TEAC_ORAC_	mg TE/g E	1164.57 ± 56.04		
	mg TE/g PM	338.89 ± 16.31	-^na^	-^na^

PM: plant material (*I. salicina*); E: extract; TPC: total phenolic content; GAE: gallic acid equivalents; TFC: total flavonoid content; QE: quercetin equivalents; CUPRAC: cupric ion reducing antioxidant capacity; TE: Trolox equivalents; TEAC: Trolox equivalent antioxidant capacity; PLE-EtOH/H_2_O_OPT:_ pressurised ethanol/water extraction under optimised conditions (82 °C, 27 min, EtOH/H_2_O 60/40% *v*/*v*). PLE-EtOH/H_2_O yields are reported as mean of three technical replicates ± SD; TPC, TFC and TEAC values are reported as mean of four technical replicates ± SD; -^na^: not applicable. The different superscript letters in the same line indicate significantly different values (*p* < 0.05) based on one-way ANOVA or an unpaired *t*-test.

**Table 4:** Qualitative phenolic compound composition analysed by UPLC-ESI/MS^2^ of *I. salicina* extract obtained under optimal PLE-EtOH/H_2_O conditions (82 °C, 27 min, EtOH/H_2_O 60/40% *v*/*v*).

No	Rt (min)	Compound	MRM Transitions		Level of Identification ^3^
			Precursor Ion [M-H] ^1^	Qualifier and Quantifier Ions ^2^	
1	3.4	Quinic acid	191	**173**	Level 2
2	7.2	Neochlorogenic acid (3-O-caffeoylquinic acid)	353	291; 179; **135**	Level 1
3	9.1	Caffeoyl hexose isomer	341	**179**, 137	Level 3
4	9.2	Ferulic acid	193	178	Level 2
5	10.2	Caffeoyl hexose isomer	341	**179;** 137	Level 3
6	10.8	Caffeoyl hexose isomer	341	**179;** 137	Level 3
7	11.05	Chlorogenic acid (5-O-caffeoylquinic acid)	353	**191;** 179; 135	Level 1
8	13.9	p-Coumaroylquinic acid isomer	337	191; 173;163	Level 3
9	14.3	p-Coumaroylquinic acid isomer	337	191; 173;163	Level 3
10	17.7	3,4-di-O-caffeoylquinic acid	515	353; 335; **191;** 179; 173	Level 2
11	18	3,5-di-O-caffeoylquinic acid	515	353; 335; **191;** 179; 173	Level 2
12	18.3	Cryptochlorogenic acid (4-O-caffeoylquinic acid)	353	**179;** 135	Level 2
13	18.8	1,5-di-O-caffeoylquinic acid	515	353, 335, **191**, 179,173	Level 2
14	19.1	Caffeoylfeuloyl quinic acid	529	367; 353; **191**; 161	Level 2
15	19.3	Nepitrin (nepetin 7-O-glucoside)	477	**315**, 299	Level 2
16	19.6	Coumaroyl-caffeoylquinic acid	499	173, 163, **191**	Level 2
17	19.6	Kaempferol-glucoside	447	285; **284**	Level 2
18	20	Caffeoyl hexose isomer	341	179, 135	Level 3
19	20	Caffeoyl-(salicyl)-hexoside	461	323,179	Level 3
20	20.1	Quercetin-hexoside	463	301; **300**	Level 3
21	20.3	Caffeoyl-coumaroylquinic acid	499	353, 337, 191, 173, **163**	Level 2
22	20.5	Nepetin (6-methoxyluteolin)	315	301; **300**	Level 2
23	20.6	Isorhamnetin hexoside	477	315, **299**	Level 3
24	20.6	Rutin (quercetin 3-O-rutinoside)	609	301	Level 2
25	20.6	4,5-di-O-caffeoylquinic acid	515	353, 335, **191**, 179,173	Level 2
26	20.7	Isoquercitrin (quercetin 3-O-glucoside)	463	301; **300**	Level 1
27	21	Coumaroyl-caffeoylquinic acid	499	337,191,173, 163	Level 2
28	21.3	Coumaroylquinic acid	483	337; 319; 191; **163**	Level 2
29	21.5	Caffeoylfeuloyl quinic acid	529	367; 353; **191**; 161	Level 2
30	22.3	Coumaroylquinic acid	483	337; 319; 191; **163**	Level 2
31	22.4	Kaempferol-glucoside (astragalin)	447	285; **284**	Level 2
32	22.6	Caffeoyl-coumaroylquinic acid isomer	499	173, 337, 353 163,	Level 3
33	22.8	Coumaroylquinic acid isomer	483	337; 319; **191**; 163	Level 3
34	23.05	Coumaroyl-caffeoylquinic acid isomer	499	353, 173, 191	Level 3
35	23.1	Caffeoylfeuloyl quinic acid isomer	529	367; 353; **191**; 161	Level 3
36	23.7	Caffeoyl-coumaroylquinic acid isomer	499	**173**	Level 3
37	24.0	Coumaroylquinic acid isomer	483	337; 319; 191; **163**	Level 3
38	24.7	Caffeoylfeuloyl quinic acid isomer	529	367; 353; **191**; 161	Level 3
39	25.7	Chrysoeriol	299	284	Level 2
40	25.8	Jaceosidin	329	**314**, 299	Level 2

Rt: retention time in MS; UPLC-ESI/MS^2^: ultraperformance liquid chromatography-electrospray tandem mass spectrometry; ^1^ [M-H]: negative ESI mode; ^2^ the quantifier ion is shown in bold; ^3^ level of identification based on Metabolomics Standards Initiative (MSI) guidelines [33]: Level 1—compounds identified by matching retention times and MRM transitions with chemical reference standards; Level 2—putatively annotated compounds, based on accurate mass, MS/MS fragmentation patterns, and comparison with literature data without chemical reference standards (tentative identification); Level 3—putatively characterised compound classes (tentative identification).

**Table 5:** The content of chlorogenic acid and its dicaffeoyl derivatives of *I. salicina* polar extract obtained under optimal PLE-EtOH/H_2_O conditions (82 °C, 27 min, EtOH/H_2_O 60/40% *v*/*v*).

Compound *	PLE-EtOH/H_2_O_OPT_ Extract	
	mg/g E	mg/g PM
Chlorogenic acid	96.92 ± 1.55 ^d^	28.21 ± 0.45 ^d^
3,4 Dicaffeoyl ester	6.54 ± 0.27 ^a^	1.90 ± 0.08 ^a^
3,5 Dicaffeoyl ester	66.60 ± 0.79 ^c^	19.38 ± 0.23 ^c^
1,5 Dicaffeoyl ester	63.61 ± 2.38 ^c^	18.51 ± 0.69 ^c^
4,5 Dicaffeoyl ester	18.43 ± 0.93 ^b^	5.36 ± 0.27 ^b^
Total:	252.11 ± 4.98	73.36 ± 1.45

PM: plant material (*I. salicina*); E: extract; PLE-EtOH/H_2_O_OPT:_ pressurised ethanol/water extraction under optimised conditions (82 °C, 27 min, EtOH/H_2_O 60/40% *v*/*v*); *: compounds quantified based on chlorogenic acid. Amounts are reported as the mean of three technical replicates ± SD. The different superscript letters in the same column indicate significantly different values (*p* < 0.05) based on one-way ANOVA or an unpaired *t*-test.

## Data Availability

The original contributions presented in this study are included in the article/Supplementary Material. Further inquiries can be directed to the corresponding author.

## Supplementary Materials

The following supporting information can be downloaded at: https://www.mdpi.com/article/10.3390/antiox15040466/s1, Table S1: Flow and block simulation specifications for Aspen Plus^®^ modelling of polar constituent extraction from *I. salicina* by PLE-EtOH/H_2_O; Table S2: Central composite design matrix and observed response values (per gram of extract) of polar constituent extraction from *I. salicina* by PLE-EtOH/H_2_O; Table S3: Analysis of correlation between *I. salicina* PLE-EtOH/H_2_O extract yield, TPC, TFC, TEAC_CUPRAC,_ TEAC_ABTS,_ and TEAC_ORAC_ values; Table S4: Fit statistics parameters of the models of *I. salicina* PLE-EtOH/H_2_O extraction optimisation; Table S5: ANOVA of the regression parameters of the *I. salicina* PLE-EtOH/H_2_O models for extract yield, TPC, TFC and TEAC values in CUPRAC, ABTS and ORAC assays; Table S6: First- and second-order polynomial regression equations of the *I. salicina* PLE-EtOH/H_2_O models for extract yield, TPC, TFC, and TEAC values in CUPRAC, ABTS, and ORAC assays; Table S7: Confirmation of the PLE-EtOH/H_2_O models for the extraction of *I. salicina* under the optimal conditions (82 °C, 27 min, 60/40% *v*/*v*); Table S8: Sun protection factors (SPFs) and UV-B absorption (%) of chlorogenic acid and *I. salicina* PLE-EtOH/H_2_O extract under the optimal conditions (82 °C, 27 min, 60/40% *v*/*v*); Figure S1: PLE-EtOH/H_2_O model of *I. salicina* extraction in Aspen Plus^®^: 1—pump; 2—preheater; 3—extractor; 4—filter; 5—flash evaporator; Figure S2: UV–vis spectra of *I. salicina* SE-EtOH, PLE-EtOH, PLE-EtOH/H_2_O-1, PLE-EtOH/H_2_O-2 and PLE-H_2_O extracts at 50 µg/mL concentration; Figure S3: Predicted and actual values of the *I. salicina*: (A) PLE-EtOH/H_2_O extract yield (g/100 g PM); (B,C) total phenolic content (TPC, mg GAE/g E and PM); (D,E) cupric ion reducing antioxidant capacity (TEAC_CUPRAC_, mg TE/g E and PM); (F,G) ABTS^+^-radical scavenging capacity (TEAC_ABTS_, mg TE/g E and PM); (I,J) cupric oxygen radical scavenging capacity (TEAC_ORAC_, mg TE/g E and PM); (K,L) total flavonoid content (TFC, mg QE/g E and PM); Figure S4: Response surface 3D plots showing the effects PLE-EtOH/H_2_O temperature (T), time (τ), and EtOH/H_2_O ratio (%, *v*/*v*) and their interactions on the *I. salicina*: (A) PLE-EtOH/H_2_O extract yield (g/100 g PM); (B,C) total phenolic content (TPC, mg GAE/g E and PM); (D) cupric ionreducing antioxidant capacity (TEAC_CUPRAC_, mg TE/g PM); (E,F) total flavonoid content (TFC, mg QE/g E and PM); Figure S5: Solvent mass fraction in *I. salicina* PLE-EtOH/H_2_O extract after evaporation as a function of temperature (A) and pressure (B) predicted by Aspen Plus^®^ model; Figure S6: Variation of flash evaporator duty (kW) as a function of evaporation temperature and pressure in the Aspen Plus^®^ simulation of *I. salicina* PLE-EtOH/H_2_O process.
