# Supplementary material for: Aspen Plus®-Validated CCD–RSM Optimisation of Pressurised Ethanol/Water Extraction for Sustainable Recovery of Antioxidant and Photoprotective Constituents from Inula salicina L
Source: Antioxidants (Basel). 2026 Apr 9;15(4):466. doi: 10.3390/antiox15040466 (PMC13113096; doi:10.3390/antiox15040466)
Supplement: Supplementary file 1 [file antioxidants-15-00466-s001.zip › antioxidants-4202007-supplementary.pdf]

Aspen Plus®-Validated CCD–RSM Optimisation of Pressurised Ethanol/Water Extraction for Sustainable Recovery of Antioxidant and Photoprotective Constituents from *Inula salicina* L.

Marius Užupis [1], Michail Syrpas [1], Andrius Jaskūnas [2], Petras Rimantas Venskutonis [1] and Vaida Kitrytė-Syrpa [1,*]

1 Department of Food Science and Technology, Kaunas University of Technology, Radvilėnų Rd. 19, LT-50254 Kaunas, Lithuania; marius.uzupis@ktu.lt (M.U.); michail.syrpas@ktu.lt (M.S.); rimas.venskutonis@ktu.lt (P.R.V.)

2 Department of Physical and Inorganic Chemistry, Kaunas University of Technology, Radvilėnų Rd. 19, LT-50254 Kaunas, Lithuania; andrius.jaskunas@ktu.lt

* Correspondence: vaida.kitryte@ktu.lt

Supplementary Material, Materials and Methods

S1. Standards and reagents

Ethanol (96 %) of agricultural origin was purchased from UAB "Vilniaus degtinė", Vilnius, Lithuania; sodium carbonate (Na_2CO_3, 98 %) from Chempur, Poland; 2M Folin-Ciocalteu's reagent, 2,9-dimethyl-1,10-phenanthroline (Neocuproine, ≥ 98%), methanol (≥ 99.9%), 2,2′-azino-bis(3-ethylbenzthiazoline-6-sulfonic acid) diammonium salt (ABTS reagent, ≥ 98%), 6-hydroxy 2,5,7,8-tetramethylchroman-2-carboxylic acid (Trolox, 97%), 2,2′-azobis(2-methylpropionamidine) dihydrochloride (AAPH reagent, 97%), 3',6'-dihydroxyspiro[isobenzofuran1(3H),9'-[9H]xanthene)-3-one (Fluorescein, >95%), analytical grade standards of gallic acid, chlorogenic acid and quercetin from Sigma-Aldrich, Germany; aluminium chloride ($AlCl_3$), sodium carbonate (Na_2CO_3), sodium chloride (NaCl), potassium dihydrophosphate (KH_2PO_4), sodium hydrophosphate (Na_2HPO_4), potassium chloride (KCl), ammonium acetate (CH_3COONH_4) from Reachem, Slovakia; potassium persulfate ($K_2S_2O_8$) from Lach-Ner, Neratovice, Czech Republic; diatomaceous earth (SiO_2, 100 %), copper chloride dihydrate ($CuCl_2•H_2O$) from Thermo Fisher Scientific, Germany. All solvents were of analytical and HPLC-grade.

S2. *In vitro* antioxidant activity assessment of *I. salicina* extracts and plant material before and after extraction

Total phenolic content (TPC) by Folin-Ciocalteu's (FC) assay. For the analysis, 150 μL of *I. salicina* extract (0.25 mg/mL) or corresponding blank were mixed with 750 μL of FC reagent (2 M), previously diluted with distilled H_2O (1:9, v/v), and after 3 min, 600 μL of Na_2CO_3 solution (75 g/L) was added, the mixture was left in the dark for 2 h. The absorbance was measured at 760 nm. TPC was expressed as gallic acid equivalents (mg GAE/g E and PM) using dose-response curve for gallic acid (0-80 μg/mL; y = 0.0127x + 0.0143; R^2 = 0,9996).

For the QUENCHER approach, 10 mg of plant material before and after extraction (particle size 0.2 mm, previously diluted with microcrystalline cellulose) or microcrystalline cellulose (blank) were mixed with 150 μL dist. H_2O, 750 μL FC reagent, and 600 μL Na_2CO_3 (75 g/L), vortexed for 15 sec, shaken at 250 rpm for 2 h in the dark, centrifuged at 4500 rpm for 5 min and the absorbance of the supernatant was measured at 760 nm. TPC was expressed as mg GAE/g PM using dose-response curve for gallic acid (y = 0.01x + 0.0182; R^2 = 0.9971).

Total flavonoid content (TFC). For the analysis, 500 μL of *I. salicina* extract (0.5 mg/mL) were combined with 500 μL of 2% aluminium trichloride ($AlCl_3$) solution or 500 μL EtOH (blank). The mixture was allowed to stand at room temperature for 10 min with intermittent shaking and the absorbance of the mixture was measured at 415 nm against a blank sample without aluminium chloride using a GENESYS 150 UV-vis spectrophotometer (Thermo Fisher Scientific, Waltham, MA, USA). The TFC was expressed as quercetin equivalents (mg QE/g E and PM) using a using dose-response of quercetin (0–50 mg/l; y = 0.0356x + 0.0249; R^2 = 0.999).

Cupric ion reducing antioxidant capacity (CUPRAC). Prior to the analysis, 1 mM $CuCl_2$ solution (0.4262 ± 0.001 g of $CuCl_2{\cdot}2H_2O$ dissolved in dist. H_2O, diluted to 250 mL), 7.5 mM neocuproine solution (0.039 ± 0.001 g of neocuproine dissolved in 96% EtOH, diluted to 25 mL) and NH_4Ac buffer (19.27 ± 0.001 g of NH_4Ac dissolved in distilled H_2O, diluted to 250 mL; pH 7) were prepared. First, 400 μL of $CuCl_2$ (1 mM), 400 μL of neocuproine (7.5 mM), and 400 μL of NH_4Ac buffer (pH 7) were mixed with 400 μL of *I.salicina* extract (0.03 mg/mL) or corresponding blank. The mixture was left in dark for 30 min, after which the absorbance was measured at 450 nm. The results were expressed as Trolox equivalent antioxidant capacity for CUPRAC ($TEAC_{CUPRAC}$, mg TE/g E and PM) using dose-response curve for Trolox (0-200 μmol/L; y = 0027x – 0.0033; R^2 = 0.9955)

For the QUENCHER approach, 10 mg of plant material before and after extraction (particle size 0.2 mm, previously diluted with microcrystalline cellulose) or microcrystalline cellulose (blank) were mixed with 400 μL of distilled H_2O, 400 μL $CuCl_2$, 400 μL neocuproine, and 400 μL NH_4Ac buffer, vortexed for 15 sec, shaken at 250 rpm for 2 h in the dark, centrifuged at 4500 rpm for 5 min and the absorbance of the supernatant was measured at 450 nm. The results were expressed as $TEAC_{CUPRAC}$ (mg TE/g PM) using dose-response curves for Trolox (y = 0.0031x + 0.0488; R^2 = 0.9994).

$ABTS^{\bullet+}$ scavenging capacity. Firstly, solution of phosphate buffer saline (PBS) (75 mmol/L; pH 7.4) was prepared: 8.18 g NaCl, 0.27 g KH_2PO_4, 1.42 g Na_2HPO_4 and 0.15 g KCl in 1 L of ultra-pure water. To prepare the $ABTS^{\bullet+}$ solution, 50 mL of ABTS (2 mmol/L PBS) was mixed with 200 μl $K_2S_2O_8$ (80 mmol/L), kept in dark for 16 h before use. To prepare the working solution, the $ABTS^{\bullet+}$ solution was diluted with PBS to obtain the absorbance of AU 0.700 ± 0.010 at 734 nm. The procedure followed adding 25 μL of *I.salicina* extract (0.25 mg/mL) or corresponding blank to 1500 μL working $ABTS^{\bullet+}$ solution, then the mixtures were kept in the dark for 2 h, finally the absorbance was measured at 734 nm. The results were expressed as $TEAC_{ABTS}$ (mg TE/g E and PM), using dose-response curves for Trolox (250-1250 μmol/L; y = 0.0709x – 2.9503; R^2 = 0.999).

For the QUENCHER approach, 10 mg of plant material before and after extraction (particle size 0.2 mm, previously diluted with microcrystalline cellulose) or microcrystalline cellulose (blank) were mixed with 25 μL of MeOH and 1500 μL working radical solution, vortexed for 15 s, shaken at 250 rpm for 2 h in the dark, centrifuged (4500 rpm, 5 min). The results were expressed as $TEAC_{ABTS}$ (mg TE/g PM) using dose-response curves for Trolox (y = 0.0786x – 3.4662; R^2 = 0.9989).

Oxygen radical absorbance capacity (ORAC). For the analysis, 25 μL of *I.salicina* extract (0.06 mg/mL) or corresponding blank was mixed with 150 μL of fluorescein solution (14 μmol/L) in 96-well black opaque microplates. After pre-incubation (15 min at 37°C), 25 μL of AAPH solution (240 mmol/L) was added rapidly, then the fluorescence was recorded at each cycle (1 min ×1.1, a total of 120 cycles) using 485-P excitation and 520-P emission filters in FLUOstar Omega reader (BMG Labtech, Offenburg, Germany). The area under the fluorescence decay curve (AUC) was calculated from the raw data, in Excel 2016 (Microsoft, Roselle, IL) as

$$AUC = 1 + \sum_{i=1}^{i=150} f_i / f_0$$

f_0 – the initial fluorescence reading at 0 (zero) min; f_i – the fluorescence reading at time *i*.

The results were expressed as $TEAC_{ORAC}$ (mg TE/g E and PM) using dose-response curves for Trolox (50-500 μmol/L; y = 0.1464x - 1,1941; R^2 = 0.998).

Supplementary Material, Tables

Table S1. Flow and block simulation specifications for Aspen Plus® modelling of polar constituent extraction from *I. salicina* by PLE-EtOH/H_2O

**Stream/Process**		**Input specification**			**Conditions**
		Compound	Mass flow, kg/h	Block	
Stream	PM*	Quercetin	0.012		P = 1 atm, T = 20 °C
	PM *	Gallic acid	0.080		P = 1 atm, T = 20 °C
	PM *	Trolox	0.230		P = 1 atm, T = 20 °C
	PM *	Insoluble matter	0.678		P = 1 atm, T = 20 °C
	EtOH**		11.434		P = 1 atm, T = 20 °C
	H_2O**		9.606		P = 1 atm, T = 20 °C
Process	Extraction	RCSTR		Kinetic data	P = 10.3 MPa, T = 40-100 °C
	Filtering	CfFilter		Fractions of solids and liquid	P = 10.3 MPa, T = 40-100 °C
	Evaporation	Separator		Flash temperature and pressure	P = 10-200 mbar, T = 45-65 °C

*Total mass flow of PM is 1 kg/h, modelled as a mixture of four components, which initial amounts were calculated based on experimental conditions (Table 2) assuming, that around 10 % of maximum content remain unextracted in the insoluble fraction. ** For modelling purposes, the mass flow of EtOH was upscaled from the laboratory procedure in which 3 g of PM were extracted with 80 mL of EtOH/H_2O, using the determined optimal 60/40 (% v/v) EtOH/H_2O ratio.

Table S2. Central composite design matrix and observed response values (per gram of extract) of polar constituent extraction from *I. salicina* by PLE-EtOH/H_2O

**CCD**		**PLE-EtOH/H_2O parameters**			**RF_2 TPC**	**RF_4 $TEAC_{CUPRAC}$**	**RF_6 $TEAC_{ABTS}$**	**RF_8 $TEAC_{ORAC}$**	**RF_{10} TFC**
Run No.	Space Type	T, ºC	τ, min	EtOH/H_2O, % v/v	mg GAE/g E	mg TE/g E	mg TE/g E	mg TE/g E	mg QE/g E
1	Central	70	30	50/50	236.53 ± 3.08	1448.56 ± 133.31	916.23 ± 39.24	1106.17 ± 77.20	32.62 ± 0.50
2	Factorial	40	15	20/80	206.03 ± 2.86	1160.83 ± 28.04	647.88 ± 56.40	1027.65 ± 35.47	28.65 ± 0.04
3	Central	70	30	50/50	239.69 ± 3.60	1357.60 ± 35.18	913.52 ± 7.42	1085.23 ± 24.31	29.84 ± 0.48
4	Central	70	30	50/50	246.97 ± 4.43	1444.40 ± 26.04	898.70 ± 45.67	1108.01 ± 33.45	31.76 ± 0.84
5	Axial	70	30	20/80	219.45 ± 9.33	1250.57 ± 23.06	763.53 ± 19.11	1121.00 ± 34.78	28.81 ± 0.63
6	Central	70	30	50/50	249.04 ± 5.93	1442.49 ± 57.34	897.01 ± 45.23	1153.11 ± 22.36	32.45 ± 0.81
7	Central	70	30	50/50	231.66 ± 4.00	1333.34 ± 31.63	861.48 ± 30.31	1171.25 ± 29.12	32.74 ± 0.34
8	Axial	100	30	50/50	235.23 ± 9.99	1362.88 ± 35.93	877.32 ± 22.34	1118.14 ± 47.35	31.06 ± 0.53
9	Axial	70	15	50/50	233.35 ± 3.95	1324.80 ± 18.39	843.02 ± 21.81	1133.98 ± 95.99	31.63 ± 0.73
10	Factorial	40	15	80/20	239.76 ± 5.12	1326.25 ± 45.57	802.36 ± 52.36	1141.98 ± 20.78	31.13 ± 0.98
11	Central	70	30	50/50	239.66 ± 4.19	1439.66 ± 38.88	863.91 ± 58.34	1091.96 ± 83.36	31.62 ± 0.81
12	Axial	70	30	80/20	244.28 ± 2.99	1420.19 ± 18.75	844.61 ± 48.57	1134.83 ± 19.04	35.54 ± 0.41
13	Factorial	100	45	80/20	269.36 ± 2.28	1541.81 ± 15.03	818.18 ± 16.35	1124.35 ± 41.00	37.17 ± 1.59
14	Axial	40	30	50/50	231.03 ± 2.25	1265.44 ± 31.23	840.07 ± 35.03	1103.38 ± 63.06	32.63 ± 0.46
15	Factorial	100	15	80/20	240.80 ± 3.17	1392.61 ± 48.80	815.14 ± 40.84	1155.28 ± 20.58	34.33 ± 0.56
16	Factorial	100	45	20/80	222.78 ± 2.06	1258.36 ± 7.65	773.61 ± 29.16	1029.67 ± 82.80	27.56 ± 0.28
17	Factorial	40	45	80/20	243.28 ± 1.06	1406.26 ± 70.75	927.13 ± 34.58	1206.03 ± 43.39	35.36 ± 1.09
18	Factorial	40	45	20/80	203.27 ± 4.64	1124.04 ± 101.17	779.60 ± 17.28	982.92 ± 20.48	29.32 ± 0.50
19	Factorial	100	15	20/80	220.06 ± 6.79	1265.93 ± 8.92	810.83 ± 26.43	1139.81 ± 55.48	30.29 ± 0.67
20	Axial	70	45	50/50	237.83 ± 2.49	1396.62 ± 16.98	892.14 ± 30.57	1116.62 ± 37.01	32.99 ± 0.94

EtOH/H_2O: pressurised ethanol/water extraction; PM: plant material (*I. salicina*); TPC: total phenolic content; E: extract; GAE: gallic acid equivalents; CUPRAC: cupric ion reducing antioxidant capacity; ABTS: $ABTS^+$ radical scavenging capacity; ORAC: oxygen radical absorbance capacity; QE: quercetin equivalents; RF: response factor; TFC: total flavonoid content; TE: Trolox equivalents; TEAC: Trolox equivalent antioxidant capacity. Extraction yields are reported as mean of three technical replicates ± SD; TPC and TEAC values are reported as mean of four technical replicates ± SD.

Table S3. Analysis of correlation between *I. salicina* PLE-EtOH/H_2O extract yield, TPC, TFC, TEAC$_{CUPRAC}$, TEAC$_{ABTS}$, and TEAC$_{ORAC}$ values

Response factors	RF$_1$	RF$_2$	RF$_3$	RF$_4$	RF$_5$	RF$_6$	RF$_7$	RF$_8$	RF$_9$	RF$_{10}$	RF$_{11}$
RF$_1$	1										
RF$_2$	-0.20262	1									
RF$_3$	0.92263****	0,18979	1								
RF$_4$	-0.065784	0,94596****	0,30619	1							
RF$_5$	0.89624****	0,2312	0,9902****	0,3820	1						
RF$_6$	0.072812	0,60257**	0,31239	0,6711**	0,3668	1					
RF$_7$	0.90231****	0,090639	0,94201****	0,2361	0,9422****	0,4911*	1				
RF$_8$	-0.37118	0,60632**	-0,13543	0,59038**	-0,08547	0,5539*	-0,084797	1			
RF$_9$	0.95891****	-0,033831	0,9479****	0,1062	0,9344****	0,236	0,93879****	-0,095341	1		
RF$_{10}$	-0.44751*	0,77932****	-0,1424	0,75006***	-0,08696	0,4756*	-0,18343	0,64535**	-0,27764	1	
RF$_{11}$	0.73134***	0,21352	0,81872****	0,3412	0,8262****	0,2579	0,75558***	0,016156	0,79503****	0,1280	1

PLE-EtOH/H_2O pressurised ethanol/water extraction; PM: plant material (*I. salicina*); E: extract; CUPRAC: cupric ion reducing antioxidant capacity; GAE: gallic acid equivalents; ORAC: oxygen radical absorbance capacity; RF: response factor; TE: Trolox equivalents; TEAC: Trolox equivalent antioxidant capacity; TFC: total flavonoid content; TPC: total phenolic content; QE: quercetin equivalents. RF$_1$: PLE-EtOH/H_2O extract yield, g/100 g PM; RF$_2$: TPC, mg GAE/g E; RF$_3$: TPC, mg GAE/g PM; RF$_4$: TEAC$_{CUPRAC}$, mg TE/g E; RF$_5$: TEAC$_{CUPRAC}$, mg TE/g PM; RF$_6$: TEAC$_{ABTS}$, mg TE/g E; RF$_7$: TEAC$_{ABTS}$, mg TE/g PM; RF$_8$: TEAC$_{ORAC}$, mg TE/g E; RF$_9$: TEAC$_{ORAC}$, mg TE/g PM; RF$_{10}$: TFC, mg QE/g E; RF$_{11}$: TFC, mg QE/g PM. *: correlation is significant at the $p<0.05$ level (two-tailed); **:correlation is significant at the $p<0.01$ level (two-tailed); ***: correlation is significant at the $p< 0.001$ level (two-tailed); ****: correlation is significant at the $p< 0.0001$ level (two-tailed). GraphPad Prism 10.4.0 software (2024) was used to calculate Pearson correlation coefficients.

Table S4. Fit statistics parameters of the models of *I. salicina* PLE-EtOH/H_2O extraction optimisation

Response factors		Fit statistics parameters								
		Suggested model	Std. Dev.	Mean	C.V. %	R^2	Adjusted R^2	Predicted R^2	Adeq. Prec.	*F*-value
RF_1Yield, g/100g PM	Prior to modification	Quadratic	0.70	26.20	2.69	0.9851	0.9717	0.9159	29.4347	73.39
	After modification	Modified quadratic	0.69		2.64	0.9814	0.9728	0.9454	35.9040	114.23
RF_2 TPC, mg GAE/g E	Prior to modification	Quadratic	6.40	234.50	2.73	0.9040	0.8177	0.4452	13.6540	10.47
	After modification	Linear	7.77		3.31	0.7740	0.7317	0.6150	15.3893	18.27
RF_3 TPC, mg GAE/g PM	Prior to modification	Quadratic	2.26	61.33	3.68	0.9732	0.9490	0.8985	21.4878	40.28
	After modification	Modified quadratic	2.12		3.46	0.9715	0.9548	0.9322	24.8522	58.37
RF_4 $TEAC_{CUPRAC}$, mg TE/g E	Prior to modification	Quadratic	45.80	1348.13	3.40	0.8977	0.8057	0.7208	12.5447	9.75
	After modification	Modified quadratic	43.20		3.20	0.8726	0.8271	0.8027	16.1272	19.17
RF_5 $TEAC_{CUPRAC}$, mg TE/g PM	Prior to modification	Quadratic	13.57	352.98	3.84	0.9745	0.9515	0.8988	21.7348	42.40
	After modification	Modified quadratic	14.27		4.04	0.9633	0.9463	0.9019	24.7643	56.83
RF_6 $TEAC_{ABTS}$, mg TE/g E	Prior to modification	Quadratic	19.80	841.09	2.35	0.9553	0.9150	0.8001	19.7598	23.73
	After modification	Modified quadratic	20.41		2.43	0.9382	0.9096	0.8780	22.9044	32.87
$RF_7$$TEAC_{ABTS}$, mg TE/g PM	Prior to modification	Quadratic	9.29	220.73	4.21	0.9734	0.9494	0.8278	21.2890	40.62
	After modification	Modified quadratic	9.17		4.15	0.9637	0.9507	0.9180	27.2618	74.27
RF_8 $TEAC_{ORAC}$, mg TE/g E	Prior to modification	Quadratic	32.19	1112.57	2.89	0.7956	0.6116	0.1710	8.6163	4.32
	After modification	2FI	30.70		2.76	0.7583	0.6467	0.2384	10.7983	6.80
RF_9 $TEAC_{ORAC}$, mg TE/g PM	Prior to modification	Quadratic	10.33	290.86	3.55	0.9723	0.9473	0.9457	20.9371	38.94
	After modification	Modified quadratic	10.13		3.48	0.9626	0.9493	0.9333	25.4199	72.13
RF_{10} TFC, mg QE/g E	Prior to modification	Quadratic	1.02	31.88	3.20	0.9103	0.8295	0.5733	12.9509	11.27
	After modification	2FI	0.9108		2.86	0.9071	0.8642	0.7405	17.3435	21.15
RF_{11} TFC, mg QE/g PM	Prior to modification	Quadratic	0.41	8.44	4.85	0.9486	0.9024	0.5419	17.2834	20.52
	After modification	Modified quadratic	0.45		5.31	0.9202	0.8833	0.7387	19.4282	24.97

PLE-EtOH/H_2O pressurised ethanol/water extraction; CV: coefficient of variation; PM: plant material (*I. salicina*); E: extract; CUPRAC: cupric ion reducing antioxidant capacity; GAE: gallic acid equivalents; ORAC: oxygen radical absorbance capacity; RF: response factor; TE: Trolox equivalents; TEAC: Trolox equivalent antioxidant capacity; TFC: total flavonoid content; TPC: total phenolic content; QE: quercetin equivalents; QE: quercetin equivalents; R^2: coefficient of determination; Std. Dev.: standard deviation; 2FI: two-factor interaction.

Table S5. ANOVA of the regression parameters of the *I. salicina* PLE-EtOH/H_2O models for extract yield, TPC, TFC and TEAC values in CUPRAC, ABTS and ORAC assays

**Source**	**Model prior to modification**					**Model after modification**				
	SS	df	MS	F-value	p-value	SS	df	MS	F-value	p-value
				**RF_1: yield, g/100g PM**						
Model	327.97	9	36.44	73.39	< 0.0001*	326.74	6	54.46	114.23	< 0.0001*
T-Temperature, °C	85.50	1	85.50	172.19	< 0.0001*	85.50	1	85.50	179.34	< 0.0001*
τ -Time, min	5.17	1	5.17	10.41	0.0091*	5.17	1	5.17	10.84	0.0058*
C- EtOH/H_2O ratio, % v/v	136.16	1	136.16	274.23	< 0.0001*	136.16	1	136.16	285.62	< 0.0001*
Tτ	0.7750	1	0.7750	1.56	0.2400**	7.86	1	7.86	16.49	0.0013*
TC	7.86	1	7.86	15.83	0.0026*					
τC	0.4005	1	0.4005	0.8066	0.3902**					
T^2	7.84	1	7.84	15.79	0.0026*	9.72	1	9.72	20.38	0.0006*
$τ^2$	0.0567	1	0.0567	0.1143	0.7423**					
C^2	24.07	1	24.07	48.48	< 0.0001*	29.04	1	29.04	60.92	< 0.0001*
Residual	4.97	10	0.4965			6.20	13	0.4767		
Lack of Fit	3.33	5	0.6659	2.04	0.2269**	4.56	8	0.5702	1.74	0.2801**
Pure Error	1.64	5	0.3271			1.64	5	0.3271		
Cor Total	332.94	19				332.94	19			
				**RF_2: TPC, mg GAE/g E**						
Model	3861.34	9	429.04	10.47	0.0005*	3306.00	3	1102.00	18.27	< 0.0001*
T-Temperature	420.68	1	420.68	10.26	0.0094*	420.68	1	420.68	6.97	0.0178*
τ -Time	133.37	1	133.37	3.25	0.1014**	133.37	1	133.37	2.21	0.1565**
C- EtOH/H_2O ratio, % v/v	2751.95	1	2751.95	67.15	< 0.0001*	2751.95	1	2751.95	45.62	< 0.0001*
Tτ	116.43	1	116.43	2.84	0.1228**					
TC	5.15	1	5.15	0.1257	0.7303**					
τC	128.96	1	128.96	3.15	0.1065**					
T^2	31.14	1	31.14	0.7598	0.4038**					
$τ^2$	2.25	1	2.25	0.0550	0.8194**					
C^2	58.95	1	58.95	1.44	0.2580**					
Residual	409.85	10	40.98			965.19	16	60.32		
Lack of Fit	199.84	5	39.97	0.9516	0.5211**	755.18	11	68.65	1.63	0.3065**
Pure Error	210.01	5	42.00			210.01	5	42.00		
Cor Total	4271.19	19				4271.19	19			

Table S5. Continued

Source	Model prior to modification					Model after modification				
	SS	df	MS	F-value	p-value	SS	df	MS	F-value	p-value
					**RF_3: TPC, mg GAE/g PM**					
Model	1845.72	9	205.08	40.28	< 0.0001*	1842.52	7	263.22	58.37	< 0.0001*
T-Temperature	724.54	1	724.54	142.30	< 0.0001*	724.54	1	724.54	160.67	< 0.0001*
τ -Time	63.66	1	63.66	12.50	0.0054*	63.66	1	63.66	14.12	0.0027*
C- EtOH/H_2O ratio, % v/v	198.83	1	198.83	39.05	< 0.0001*	198.83	1	198.83	44.09	< 0.0001*
Tτ	24.26	1	24.26	4.76	0.0540**	24.26	1	24.26	5.38	0.0388*
TC	42.74	1	42.74	8.39	0.0159*	42.74	1	42.74	9.48	0.0096*
τC	2.09	1	2.09	0.4107	0.5360**					
T^2	65.47	1	65.47	12.86	0.0050*	83.78	1	83.78	18.58	0.0010*
$τ^2$	1.11	1	1.11	0.2172	0.6512**					
C^2	201.70	1	201.70	39.61	< 0.0001*	247.91	1	247.91	54.98	< 0.0001*
Residual	50.92	10	5.09			54.11	12	4.51		
Lack of Fit	13.81	5	2.76	0.3720	0.8491**	17.00	7	2.43	0.3272	0.9112**
Pure Error	37.11	5	7.42			37.11	5	7.42		
Cor Total	1896.63	19				1896.63	19			
					**RF_4: $TEAC_{CUPRAC}$, mg TE/ g E**					
Model	184094.54	9	20454.95	9.75	0.0007*	178900.00	5	35787.12	19.17	< 0.0001*
T-Temperature	29027.31	1	29027.31	13.84	0.0040*	29027.31	1	29027.31	15.55	0.0015*
τ -Time	6587.95	1	6587.95	3.14	0.1067**	6587.95	1	6587.95	3.53	0.0813*
C- EtOH/H_2O ratio, % v/v	105553.02	1	105553.02	50.33	< 0.0001*	105553.02	1	105553.02	56.55	< 0.0001*
Tτ	1210.57	1	1210.57	0.5772	0.4649**					
TC	175.88	1	175.88	0.0839	0.7780**					
τC	9355.07	1	9355.07	4.46	0.0608**	9355.07	1	9355.07	5.01	0.0419*
T^2	7651.26	1	7651.26	3.65	0.0852**	28412.23	1	28412.23	15.22	0.0016*
$τ^2$	105.62	1	105.62	0.0504	0.8270**					
C^2	2733.41	1	2733.41	1.30	0.2802**					
Residual	20972.25	10	2097.23			26131.21	14	1866.52		
Lack of Fit	7750.29	5	1550.06	0.5862	0.7140**	12909.25	9	1434.36	0.5424	0.7996**
Pure Error	13221.96	5	2644.39			13221.96	5	2644.39		
Cor Total	205066.79	19				205066.79	19			

Table S5. Continued

Source	Model prior to modification					Model after modification				
	SS	df	MS	F-value	p-value	SS	df	MS	F-value	p-value
					**RF_5: $TEAC_{CUPRAC}$, mg TE/ g PM**					
Model	70259.37	9	7806.60	42.40	< 0.0001*	69452.34	6	11575.39	56.83	< 0.0001*
T-Temperature	27639.20	1	27639.20	150.13	< 0.0001*	27639.20	1	27639.20	135.69	< 0.0001*
τ -Time	2286.45	1	2286.45	12.42	0.0055*	2286.45	1	2286.45	11.22	0.0052*
C- EtOH/H_2O ratio, % v/v	5378.22	1	5378.22	29.21	0.0003*	5378.22	1	5378.22	26.40	0.0002*
Tτ	539.40	1	539.40	2.93	0.1177**					
TC	1312.51	1	1312.51	7.13	0.0235*	1312.51	1	1312.51	6.44	0.0247*
τC	225.25	1	225.25	1.22	0.2946**					
T^2	3583.02	1	3583.02	19.46	0.0013*	4516.36	1	4516.36	22.17	0.0004*
$τ^2$	42.39	1	42.39	0.2302	0.6417**					
C^2	7221.89	1	7221.89	39.23	< 0.0001*	8893.44	1	8893.44	43.66	< 0.0001*
Residual	1841.07	10	184.11			2648.11	13	203.70		
Lack of Fit	936.56	5	187.31	1.04	0.4852**	1743.59	8	217.95	1.20	0.4375**
Pure Error	904.52	5	180.90			904.52	5	180.90		
Cor Total	72100.44	19				72100.44	19			
					**RF_6: $TEAC_{ABTS}$, mg TE/ g E**					
Model	83699.00	9	9299.89	23.73	< 0.0001*	82200.83	6	13700.14	32.87	< 0.0001*
T-Temperature	961.18	1	961.18	2.45	0.1484**	961.18	1	961.18	2.31	0.1528**
τ -Time	7367.42	1	7367.42	18.80	0.0015*	7367.42	1	7367.42	17.68	0.0010*
C- EtOH/H_2O ratio, % v/v	18659.81	1	18659.81	47.60	< 0.0001*	18659.81	1	18659.81	44.77	< 0.0001*
Tτ	10561.13	1	10561.13	26.94	0.0004*	10561.13	1	10561.13	25.34	0.0002*
TC	8009.35	1	8009.35	20.43	0.0011*	8009.35	1	8009.35	19.22	0.0007*
τC	138.69	1	138.69	0.3538	0.5652**					
T^2	749.18	1	749.18	1.91	0.1969**					
$τ^2$	159.70	1	159.70	0.4074	0.5376**					
C^2	13913.81	1	13913.74	35.50	0.0001*	36641.94	1	36641.94	87.92	< 0.0001*
Residual	3919.81	10	391.98			5417.98	13	416.77		
Lack of Fit	2182.47	5	436.49	1.26	0.4042**	3680.64	8	460.08	1.32	0.3945**
Pure Error	1737.34	5	347.47			1737.34	5	347.47		
Cor Total	87618.81	19				87618.81	19			

Table S5. Continued

Source	Model prior to modification					Model after modification				
	SS	df	MS	F-value	p-value	SS	df	MS	F-value	p-value
					**RF_7: $TEAC_{ABTS}$, mg TE/ g PM**					
Model	31534.43	9	3503.83	40.62	< 0.0001*	31219.94	5	6243.99	74.27	< 0.0001*
T-Temperature	7493.91	1	7493.91	86.88	< 0.0001*	7493.91	1	7493.91	89.13	< 0.0001*
τ -Time	1338.65	1	1338.65	15.52	0.0028*	1338.65	1	1338.65	15.92	0.0013*
C- EtOH/H_2O ratio, % v/v	3871.06	1	3871.06	44.88	< 0.0001*	3871.06	1	3871.06	46.04	< 0.0001*
Tτ	237.73	1	237.73	2.76	0.1279**					
TC	16.45	1	16.45	0.1907	0.6716**					
τC	28.16	1	28.16	0.3265	0.5803**					
T^2	969.33	1	969.33	11.24	0.0073*	1287.29	1	1287.29	15.31	0.0016*
$τ^2$	32.16	1	32.16	0.3728	0.5551**					
C^2	5664.33	1	5664.33	65.67	< 0.0001*	6968.95	1	6968.95	82.89	< 0.0001*
Residual	862.55	10	86.26			1177.04	14	84.07		
Lack of Fit	570.35	5	114.07	1.95	0.2403**	884.84	9	98.32	1.68	0.2942**
Pure Error	292.20	5	58.44			292.20	5	58.44		
Cor Total	32396.98	19				32396.98	19			
					**RF_8: $TEAC_{ORAC}$, mg TE/ g E**					
Model	40315.27	9	4479.47	4.32	0.0160*	38425.34	6	6404.22	6.80	0.0020*
T-Temperature	1108.60	1	1108.60	1.07	0.3253**	1108.60	1	1108.60	1.18	0.2978**
τ -Time	1935.16	1	1935.16	1.87	0.2017**	1935.16	1	1935.16	2.05	0.1755**
C- EtOH/H_2O ratio, % v/v	21290.84	1	21290.84	20.55	0.0011*	21290.84	1	21290.84	22.59	0.0004*
Tτ	3215.62	1	3215.62	3.10	0.1086**	3215.62	1	3215.62	3.41	0.0876**
TC	6457.59	1	6457.59	6.23	0.0316*	6457.59	1	6457.59	6.85	0.0213*
τC	4417.53	1	4417.53	4.26	0.0658**	4417.53	1	4417.53	4.69	0.0496*
T^2	833.98	1	833.98	0.8050	0.3907**					
$τ^2$	22.72	1	22.72	0.0219	0.8852**					
C^2	0.1853	1	0.1853	0.0002	0.9896**					
Residual	10359.99	10	1036.00			12249.93	13	942.30		
Lack of Fit	4309.97	5	861.99	0.7124	0.6406**	6199.91	8	774.99	0.6405	0.7263**
Pure Error	6050.02	5	1210.00			6050.02	5	1210.00		
Cor Total	50675.27	19				50675.27	19			

Table S5. Continued

Source	Model prior to modification					Model after modification				
	SS	df	MS	F-value	p-value	SS	df	MS	F-value	p-value
					**RF_9: $TEAC_{ORAC}$, mg TE/ g PM**					
Model	37384,05	9	4153.78	38.94	< 0.0001*	37013.92	5	7402.78	72.13	< 0.0001*
T-Temperature	12970,08	1	12970.08	121.59	< 0.0001*	12970.08	1	12970.08	126.37	< 0.0001*
τ -Time	119,44	1	119.44	1.12	0.3149**	119.44	1	119.44	1.16	0.2989**
C- EtOH/H_2O ratio, % v/v	9094,45	1	9094.45	85.25	< 0.0001*	9094.45	1	9094.45	88.61	< 0.0001*
Tτ	12,35	1	12.35	0.1158	0.7407**					
TC	229,84	1	229.84	2.15	0.1729**					
τC	111,75	1	111.75	1.05	0.3302**					
T^2	1631,39	1	1631.39	15.29	0.0029*	2042.83	1	2042.83	19.90	0.0005*
$τ^2$	16,19	1	16.19	0.1518	0.7050**					
C^2	3273,45	1	3273.45	30.69	0.0002*	4012.66	1	4012.66	39.10	< 0.0001*
Residual	1066,74	10	106.67			1436.87	14	102.63		
Lack of Fit	121,55	5	24.31	0.1286	0.9790**	491.68	9	54.63	0.2890	0.9492**
Pure Error	945,20	5	189.04			945.20	5	189.04		
Cor Total	38450,80	19				38450.80	19			
					**RF_{10}: TFC, mg QE/ g E**					
Model	105.63	9	11.74	11.27	0.0004*	105.26	6	17.54	21.15	< 0.0001*
T-Temperature	1.10	1	1.10	1.06	0.3278**	1.10	1	1.10	1.33	0.2698**
τ -Time	4.06	1	4.06	3.90	0.0766**	4.06	1	4.06	4.89	0.0455*
C- EtOH/H_2O ratio, % v/v	83.52	1	83.52	80.21	< 0.0001*	83.52	1	83.52	100.69	< 0.0001**
Tτ	2.87	1	2.87	2.75	0.1280**	2.87	1	2.87	3.46	0.0857**
TC	3.29	1	3.29	3.16	0.1059**	3.29	1	3.29	3.97	0.0679**
τC	10.42	1	10.42	10.01	0.0101*	10.42	1	10.42	12.56	0.0036*
T^2	0.3003	1	0.3003	0.2884	0.6030**					
$τ^2$	0.0498	1	0.0498	0.0478	0.8313**					
C^2	0.0000005682	1	0.0000005682	0.0000005456	0.9994**					
Residual	10.41	10	1.04			10.78	13	0.8295		
Lack of Fit	4.57	5	0.9136	0.7815	0.6033**	4.94	8	0.6173	0.5280	0.7988**
Pure Error	5.85	5	1.17			5.85	5	1.17		
Cor Total	116.04	19				116.04	19			

Table S5. Continued

Source	Model prior to modification					Model after modification				
	SS	df	MS	F-value	p-value	SS	df	MS	F-value	p-value
				**RF_{11}: TFC, mg QE/ g PM**						
Model	31.00	9	3.44	20.52	< 0.0001*	30.07	6	5.01	24.97	< 0.0001*
T-Temperature	16.28	1	16.28	97.00	< 0.0001*	16.28	1	16.28	81.12	< 0.0001*
τ -Time	0.0410	1	0.0410	0.2440	0.6320**	0.0410	1	0.0410	0.2041	0.6589**
C- EtOH/H_2O ratio, % v/v	0.3062	1	0.3062	1.82	0.2065**	0.3063	1	0.3063	1.53	0.2386**
Tτ	1.04	1	1.04	6.22	0.0318*	1.04	1	1.04	5.20	0.0401*
TC	6.00	1	6.00	35.76	0.0001*	6.00	1	6.00	29.91	0.0001*
τC	0.1653	1	0.1653	0.9849	0.3444**					
T^2	0.7540	1	0.7540	4.49	0.0601**					
$τ^2$	0.0511	1	0.0511	0.3047	0.5931**					
C^2	2.22	1	2.22	13.23	0.0046*	6.40	1	6.40	31.86	< 0.0001*
Residual	1.68	10	0.1679			2.61	13	0.2007		
Lack of Fit	1.20	5	0.2403	2.52	0.1669**	2.13	8	0.2665	2.79	0.1365**
Pure Error	0.4771	5	0.0954			0.4771	5	0.0954		
Cor Total	32.68	19				32.68	19			

PM: plant material (*I. salicina*); CUPRAC: cupric ion reducing antioxidant capacity; *df*: degree of freedom; *F*: Fisher value; GAE: gallic acid equivalents; *MS*: mean square; ORAC: oxygen radical absorbance capacity; PLE-EtOH/H_2O: pressurised ethanol/water extraction; QE: quercetin equivalents; RF: response factor; *SS*: sum of squares; TFC: total flavonoid content; TPC: total phenolic content; TE: Trolox equivalents; TEAC: Trolox equivalent antioxidant capacity. *: significant ($p < 0.05$); **: not significant ($p > 0.05$)

Table S6. First- and second-order polynomial regression equations of the *I. salicina* PLE-EtOH/H_2O models for extract yield, TPC, TFC and TEAC values in CUPRAC, ABTS and ORAC assays

RF	Regression equations	
	**In coded factors**	**In actual factors**
**RF_1**	28.58 + 2.92×T + 0.72×τ - 3.69×C + 0.99×TC + 1.74×T^2 - 3.01×C^2	12.4697 + 0.3134×T + 0.0479×τ + 0.1346×C + 0.0011×TC - 0.0019×T^2 - 0.0033×C^2
**RF_2**	234.50 + 6.49×T + 3.65×τ + 16.59×C	184.4167 + 0.2162×T + 0.2435×τ + 0.5530×C
**RF_3**	68.29 + 8.51×T + 2.52×τ - 4.46×C + 1.74×Tτ + 2.31×TC - 5.12×T^2 - 8.80×C^2	15.6165 + 0.8352×T - 0.1027×τ + 0.6496×C + 0.0039×Tτ + 0.0026×TC - 0.0057×T^2 - 0.0098×C^2
**RF_4**	1385.82 + 53.88×T + 25.67×τ + 102.74×C + 34.20×τC – 75.38×T^2	741.1187 + 13,522×T - 2.0885×τ + 1.1459×C + 0.076×τC - 0.0838×T^2
**RF_5**	398.12 + 52.57×T + 15.12×τ - 23.19×C + 12.81×TC - 52.57×C^2	-17.3404 + 6.8848×T + 1.0081×τ + 0.0142×C – 0.0417×TC - 0.0586×C^2
**RF_6**	883.89 + 9.80×T + 27.14×τ + 43.20×C - 36.33×Tτ - 31.64×TC - 85.61×C^2	204.3348 + 4.5069×T + 7.4615×τ + 13.4127×C - 0.0807×Tτ – 0.0352×TC - 0.0951×C^2
**RF_7**	254.09 + 27.38×T + 11.57×τ - 19.67×C - 20.06×T^2 - 46.67×C^2	-38.9627 + 4.0325×T + 0.7713×τ + 4.5294×C - 0.022×T^2 - 0.0519×C^2
**RF_8**	1112.57 + 10.53×T - 13.91×τ + 46.14×C - 20.05×Tτ - 28.41T×C + 23.50×Tτ	913.1996 + 3.266×T - 0.4197×τ + 2.1813×C - 0.0446×Tτ - 0.0316×TC + 0.0522×Tτ
**RF_9**	321.20 + 36.01×T + 3.46×τ - 30.16×C - 25.27×T^2 - 35.41×C^2	44.589 + 5.1308×T + 0.2304×τ + 2.9294×C - 0.0281×T^2 - 0.0394×C^2
**RF_{10}**	31.87 + 0.33×T + 0.64×τ + 2.89×C - 0.60×Tτ + 0.64×TC + 1.14×τC	28.5134 + 0.0154×T + 0.0088×τ - 0.0296×C - 0.0013×Tτ + 0.0007×TC + 0.0025×C^2
**RF_{11}**	9.01 + 1.28×T + 0.06×τ - 0.18×C - 0.36×Tτ + 0.87×TC - 1.13×C^2	4.7376 + 0.0185×T + 0.0605×τ + 0.05246×C - 0.0008×Tτ + 0.0010×TC - 0.0013×C^2

T-temperature; τ -time; C- EtOH/H_2O ratio, % v/v; RF: response factor; RF_1: PLE-EtOH/H_2O extract yield, g/100 g PM; RF_2: TPC, mg GAE/g E; RF_3: TPC, mg GAE/g PM; RF_4: $TEAC_{CUPRAC}$, mg TE/g E; RF_5: $TEAC_{CUPRAC}$, mg TE/g PM; RF_6: $TEAC_{ABTS}$, mg TE/g E; RF_7: $TEAC_{ABTS}$, mg TE/g PM; RF_8: $TEAC_{ORAC}$, mg TE/g E; RF_9: $TEAC_{ORAC}$, mg TE/g PM; RF_{10}: TFC, mg QE/g E; RF_{11}: TFC, mg QE/g PM; PM: plant material (*I. salicina*); CUPRAC: cupric ion reducing antioxidant capacity; GAE: gallic acid equivalents; ORAC: oxygen radical absorbance capacity; TE: Trolox equivalents; TEAC: Trolox equivalent antioxidant capacity; TFC: total flavonoid content; TPC: total phenolic content.

Table S7. Confirmation of the PLE-EtOH/H_2O models for the extraction of *I. salicina* under the optimal conditions (82°C, 27 min, 60/40% v/v)

Fit statistics parameters	RF_1 Yield	RF_2 TPC	RF_3 TPC	RF_4 $TEAC_{CUPRAC}$	RF_5 $TEAC_{CUPRAC}$	RF_6 $TEAC_{ABTS}$	RF_7 $TEAC_{ABTS}$	RF_8 $TEAC_{ORAC}$	RF_9 $TEAC_{ORAC}$	RF_{10} TFC	RF_{11} TFC
Predicted mean	27.89	241.90	68.07	1422.15	398.24	885.96	247.77	1131.19	316.88	32.90	9.47
Predicted median	27.89	241.90	68.07	1422.15	398.24	885.96	247.77	1131.19	316.88	32.90	9.47
Std. Dev.	0.69	7.77	2.12	43.20	14.27	20.41	9.17	30.70	10.13	0.91	0.45
SE Pred.	0.73	8.08	2.25	45.41	15.09	21.56	9.68	31.97	10.70	0.95	0.47
95% PI (low)	26.32	224.78	63.18	1324.75	365.65	839.40	227.01	1062.12	293.94	30.85	8.45
Exp. mean	29.10	227.39	66.17	1472.91	428.62	868.73	252.80	1164.57	338.89	34.12	9.93
Exp. Std. Dev.	0.46	3.45	1.00	52.44	15.26	47.19	13.73	56.04	16.31	1.15	0.34
95% PI (high)	29.47	259.02	72.96	1519.54	430.83	932.52	268.54	1200.26	339.83	34.95	10.49

PM: plant material (*I. salicina*); CUPRAC: cupric ion reducing antioxidant capacity; Exp. Data mean: experimental data mean; GAE: gallic acid equivalents; ORAC: oxygen radical absorbance capacity; PI: prediction interval; RF: response factor; RF_1: PLE-EtOH/H_2O extract yield, g/100 g PM; RF_2: TPC, mg GAE/g E; RF_3: TPC, mg GAE/g PM; RF_4: $TEAC_{CUPRAC}$, mg TE/g E; RF_5: $TEAC_{CUPRAC}$, mg TE/g PM; RF_6: $TEAC_{ABTS}$, mg TE/g E; RF_7: $TEAC_{ABTS}$, mg TE/g PM; RF_8: $TEAC_{ORAC}$, mg TE/g E; RF_9: $TEAC_{ORAC}$, mg TE/g PM; RF_{10}: TFC, mg QE/g E; RF_{11}: TFC, mg QE/g PM; SE Pred.: standard deviation associated with the prediction of an individual observation; Std. Dev.: standard deviation; TE: Trolox equivalents; TEAC: Trolox equivalent antioxidant capacity; TFC: total flavonoid content; TPC: total phenolic content.

Table S8. Sun protection factors (SPF) and UV-B absorption (%) of chlorogenic acid and *I. salicina* PLE-EtOH/H_2O extract under the optimal conditions (82°C, 27 min, 60/40% v/v)

***I. salicina* PLE-EtOH/H_2O extract**			**Chlorogenic acid**		
Concentration (µg/mL)	SPF	UV-B absorption, %	Concentration (µg/mL)*	SPF	UV-B absorption, %
5	0.86 ± 0.03^{a}	-nd	0.49	0.31 ± 0.22^{a}	-nd
10	1.75 ± 0.01^{b}	43.01 ± 0.17^{a}	0.97	0.51 ± 0.00^{b}	-nd
25	4.35 ± 0.05^{c}	77.03 ± 0.26^{b}	2.42	1.25 ± 0.03^{c}	19.66 ± 1.98^{a}
50	8.65 ± 0.02^{d}	88.44 ± 0.03^{c}	4.85	2.44 ± 0.01^{d}	59.00 ± 0.21^{b}
75	13.07 ± 0.03^{e}	92.35 ± 0.02^{d}	7.27	3.66 ± 0.01^{e}	72.66 ± 0.07^{c}
100	16.97 ± 0.15^{f}	94.11 ± 0.05^{e}	9.69	4.78 ± 0.10^{f}	79.09 ± 0.44^{d}
150	26.02 ± 0.08^{g}	96.16 ± 0.01^{f}	14.54	7.21 ± 0.02^{g}	86.13 ± 0.03^{e}
200	34.40 ± 0.16^{h}	97.09 ± 0.01^{g}	19.38	9.61 ± 0.02^{h}	89.59 ± 0.02^{f}
250	41.35 ± 0.15^{i}	97.58 ± 0.01^{h}	24.23	11.95 ± 0.03^{i}	91.63 ± 0.02^{g}
500	47.49 ± 0.09^{j}	97.89 ± 0.00^{i}	48.46	23.95 ± 0.11^{j}	95.82 ± 0.02^{h}
1000	49.86 ± 0.00^{k}	97.99 ± 0.00^{i}	96.92	40.84 ± 0.14^{k}	97.55 ± 0.01^{i}

*: The concentration range for chlorogenic acid was selected based on its measured level in the *I. salicina* PLE EtOH/H_2O extract (96.92 mg/g E; Table 3). -nd: not detected. SPF and UV-B absorption (%) values are reported as mean of four technical replicates ± SD. The different superscript letters in the same column indicate significantly different values ($p < 0.05$; based on one-way ANOVA).

Supplementary Material, Figures

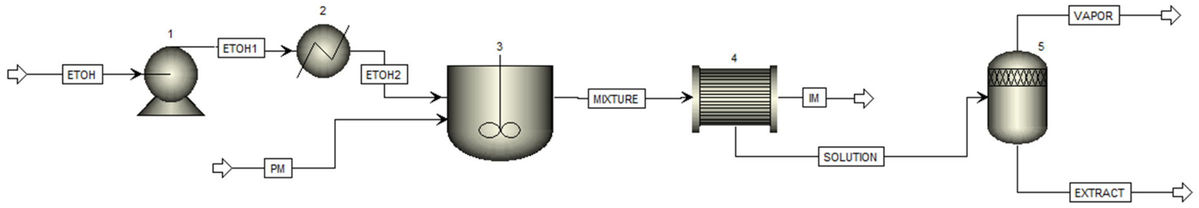

Fig. S1. PLE-EtOH/H_2O model of *I.salicina* extraction in Aspen Plus®: 1 – pump; 2 – preheater; 3 – extractor; 4 – filter; 5 – flash evaporator

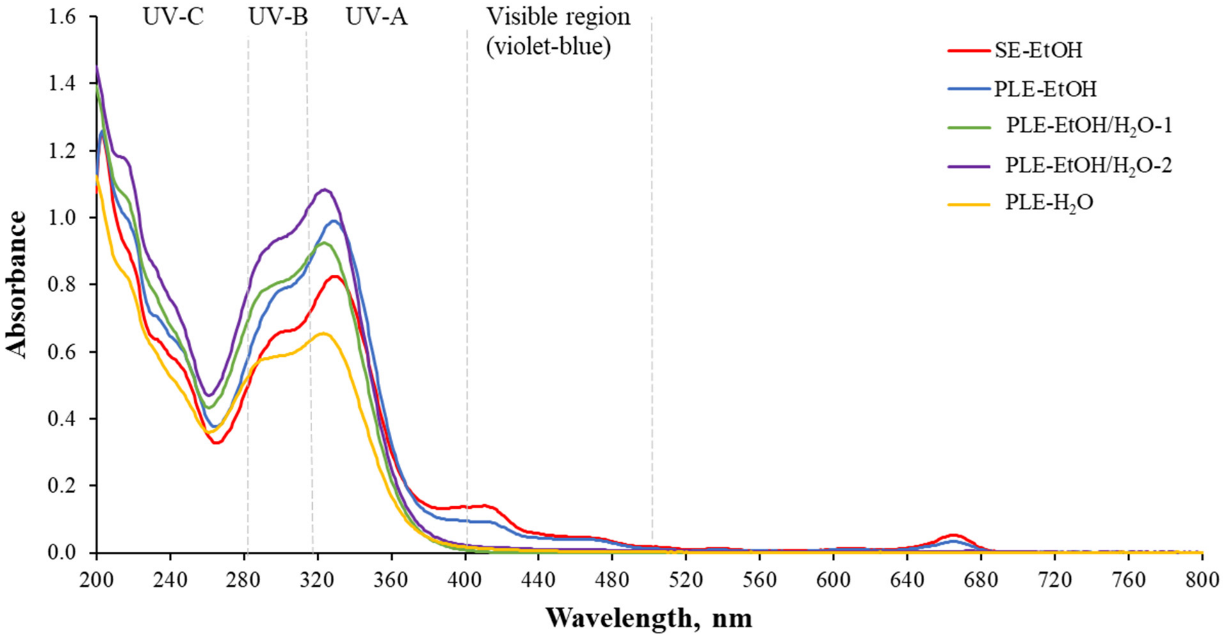

Fig. S2. UV-Vis spectra of *I. salicina* SE-EtOH, PLE-EtOH, PLE-EtOH/H_2O-1, PLE-EtOH/H_2O-2 and PLE-H_2O extracts at 50 µg/mL concentration

PLE-EtOH: (45 min, 70°C, 10.3 MPa); PLE-EtOH/H_2O-1: (45 min, 70°C, 10.3 MPa, EtOH/H_2O: 70/30 % v/v); PLE-EtOH/H_2O-2: (45 min, 70°C, 10.3 MPa, EtOH/H_2O: 50/50 % v/v); PLE-H_2O: (45 min, 110°C, 10.3 MPa); SE-EtOH: (6 h, 78°C).

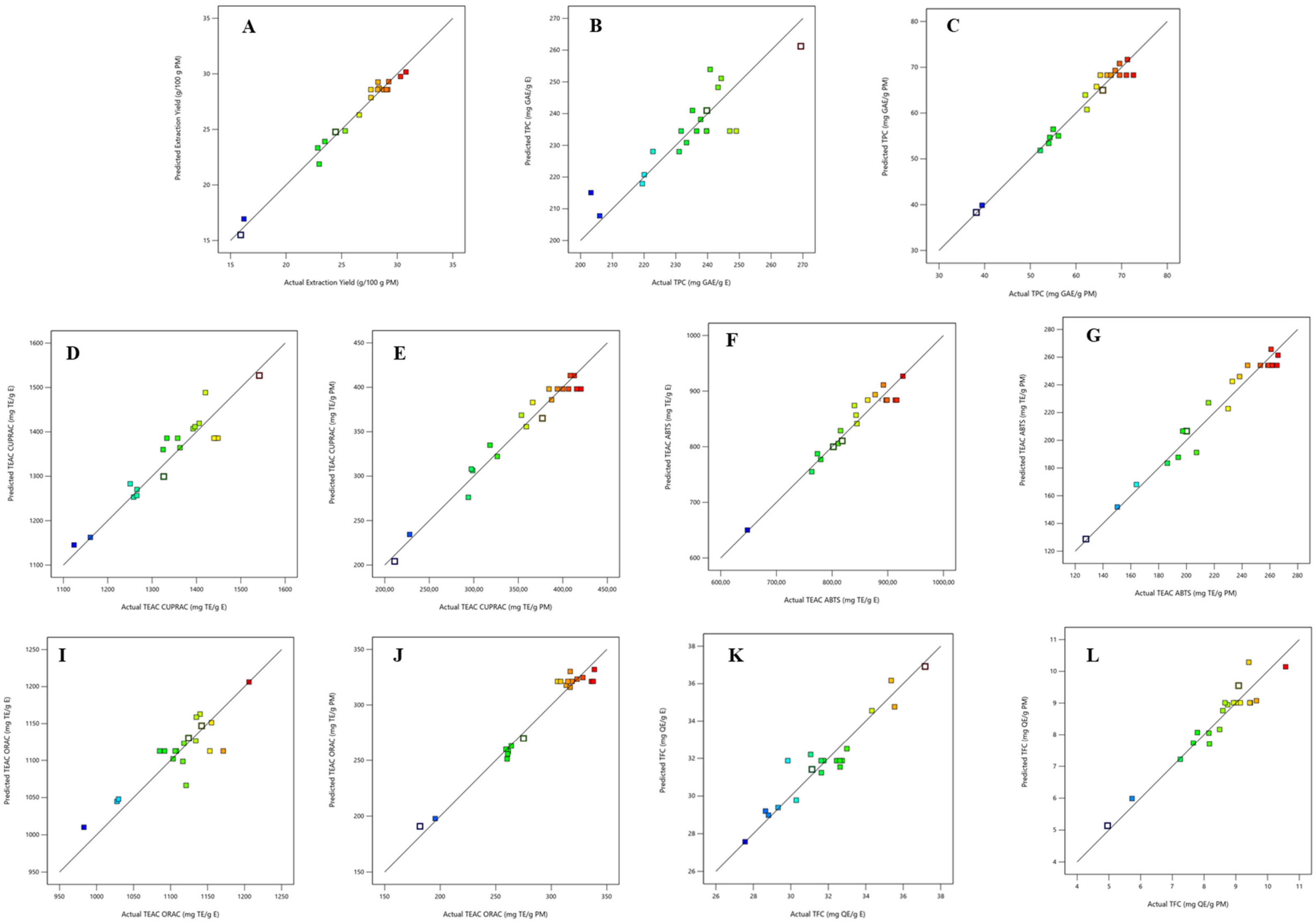

Fig. S3. Predicted and actual values of the *I.salicina*: (A) PLE-EtOH/H_2O extract yield (g/100 g PM); (B-C) total phenolic content (TPC, mg GAE/g E and PM); (D-E) cupric ion reducing antioxidant capacity ($TEAC_{CUPRAC}$, mg TE/g E and PM); (F-G) $ABTS^{+}$ radical scavenging capacity ($TEAC_{ABTS}$, mg TE/g E and PM); (I-J) cupric oxygen radical scavenging capacity ($TEAC_{ORAC}$, mg TE/g E and PM); (K-L) total flavonoid content (TFC, mg QE/g E and PM)

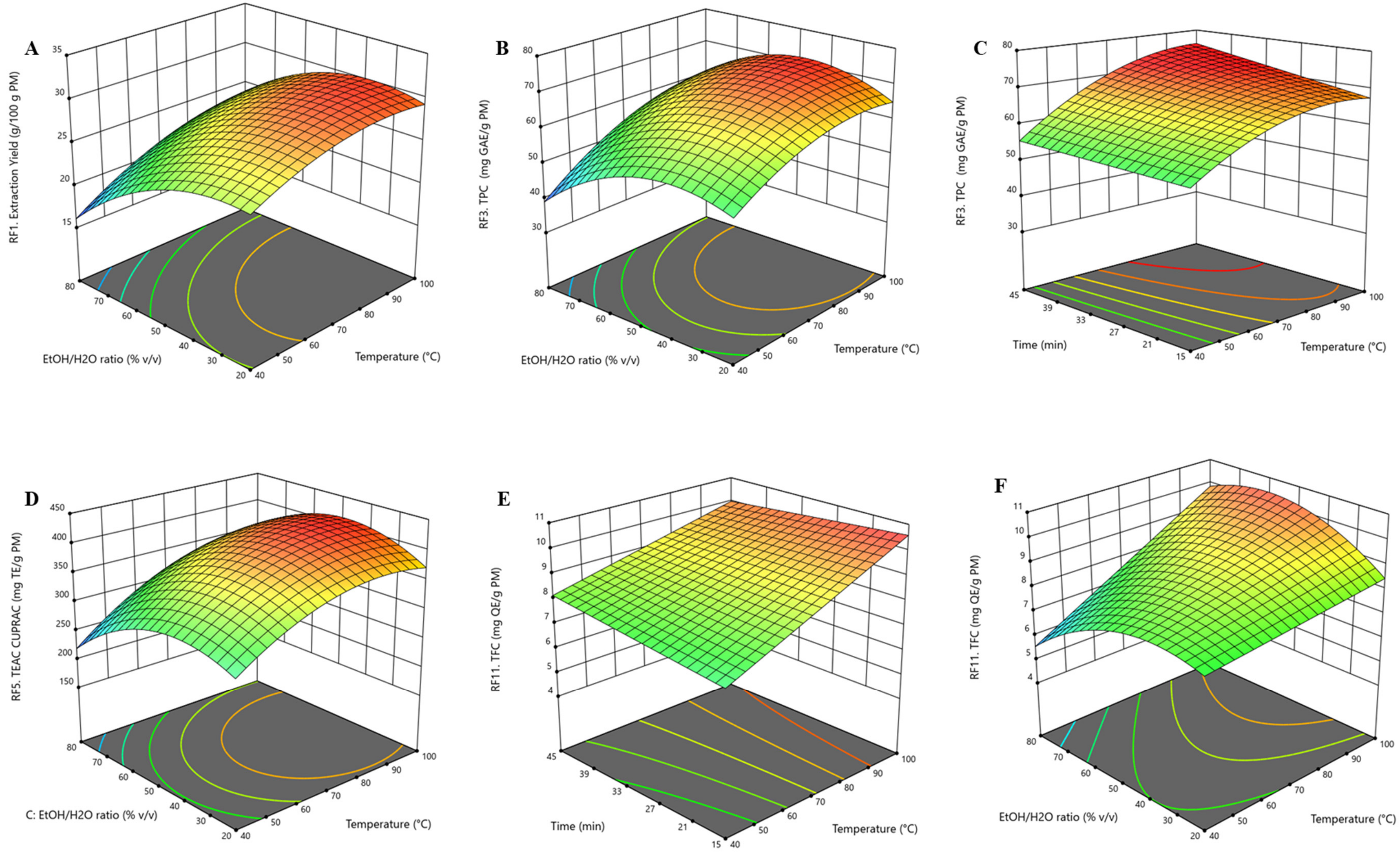

Fig. S4. Response surface 3D plots showing the effects PLE-EtOH/H_2O temperature (T), time (τ), and EtOH/H_2O ratio (% v/v) and their interactions on the *I.salicina*: (A) PLE-EtOH/H_2O extract yield (g/100 g PM); (B-C) total phenolic content (TPC, mg GAE/g E and PM); (D) cupric ion reducing antioxidant capacity ($TEAC_{CUPRAC}$, mg TE/g PM); (E-F) total flavonoid content (TFC, mg QE/g E and PM)

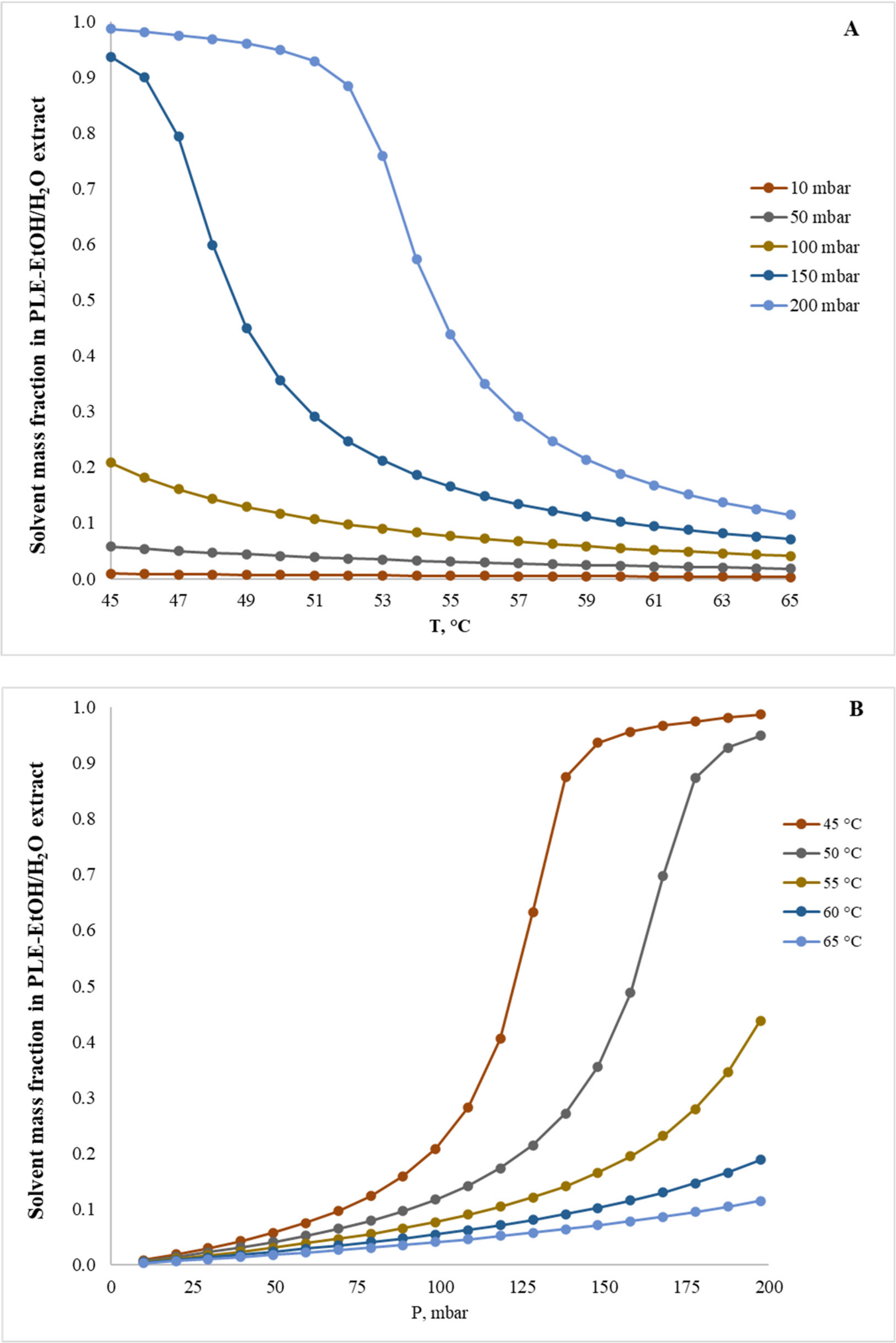

Fig. S5. Solvent mass fraction in *I.salicina* PLE-EtOH/H_2O extract after evaporation as a function of temperature (A) and pressure (B) predicted by Aspen Plus® model

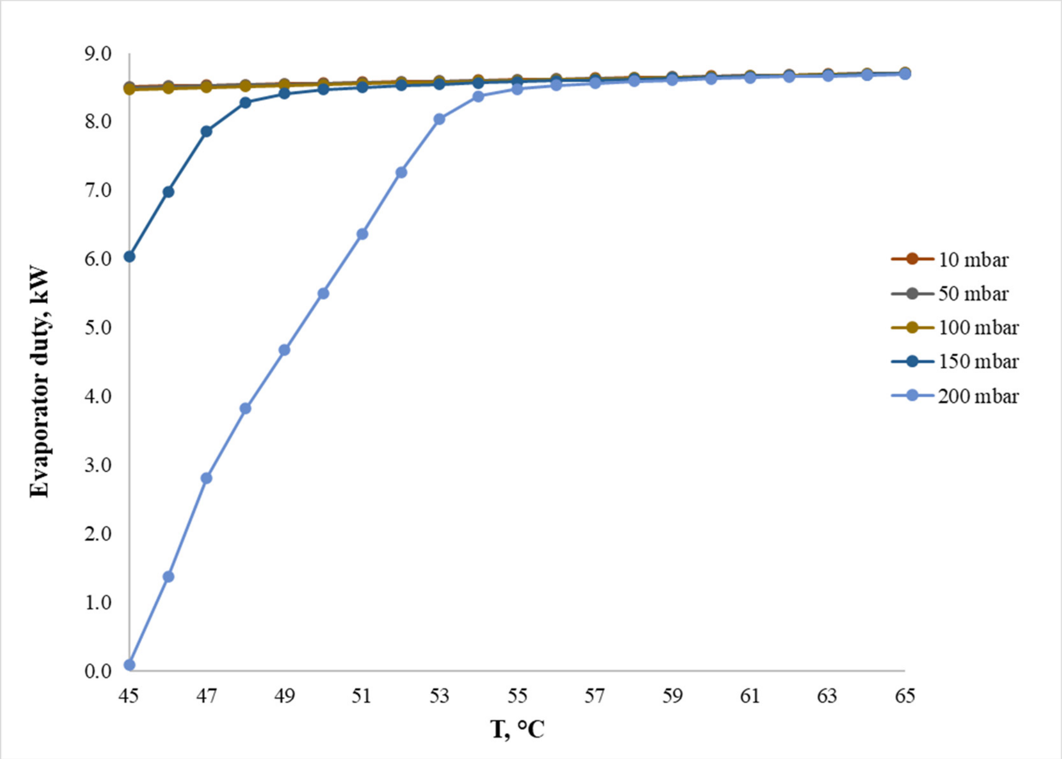

Fig. S6. Variation of flash evaporator duty (kW) as a function of on evaporation temperature and pressure in the Aspen Plus® simulation of *I. salicina* PLE-EtOH/H_2O process
